# Evolution and development of three highly specialized floral structures of bee-pollinated *Phalaenopsis* species

**DOI:** 10.1186/s13227-020-00160-z

**Published:** 2020-08-10

**Authors:** Dewi Pramanik, Nemi Dorst, Niels Meesters, Marlies Spaans, Erik Smets, Monique Welten, Barbara Gravendeel

**Affiliations:** 1 Naturalis Biodiversity Center, Endless Forms Group, Darwinweg 2, 2333 CR Leiden, The Netherlands; 2 Intitute of Biology Leiden, Leiden University, Sylviusweg 72, 2333 BE Leiden, The Netherlands; 3 Indonesian Ornamental Crops Research Institute (IOCRI), Jl. Raya Ciherang, Pacet-Cianjur, 43253 West Java Indonesia; 4 Faculty of Science and Technology, University of Applied Sciences Leiden, Zernikedreef 11, 2333 CK Leiden, The Netherlands; 5 Life Sciences, HAN University of Applied Sciences, Ruitenbergerlaan 31, 6826 CC Arnhem, The Netherlands; 6 Ecology, Evolution and Biodiversity Conservation, KU Leuven, Kasteelpark Arenberg 31, P.O. Box 2435, 3001 Heverlee, Belgium; 7 IWWR, Radboud University, Heyendaalseweg 135, 6500 GL Nijmegen, The Netherlands

**Keywords:** Callus, Mentum, Stelidia, RT-PCR, Transcriptomics

## Abstract

**Background:**

Variation in shape and size of many floral organs is related to pollinators. Evolution of such organs is driven by duplication and modification of MADS-box and MYB transcription factors. We applied a combination of micro-morphological (SEM and micro 3D-CT scanning) and molecular techniques (transcriptome and RT-PCR analysis) to understand the evolution and development of the callus, stelidia and mentum, three highly specialized floral structures of orchids involved in pollination. Early stage and mature tissues were collected from flowers of the bee-pollinated *Phalaenopsis equestris* and *Phalaenopsis pulcherrima*, two species that differ in floral morphology: *P. equestris* has a large callus but short stelidia and no mentum, whereas *P. pulcherrima* has a small callus, but long stelidia and a pronounced mentum.

**Results:**

Our results show the stelidia develop from early primordial stages, whereas the callus and mentum develop later. In combination, the micro 3D-CT scan analysis and gene expression analyses show that the callus is of mixed petaloid-staminodial origin, the stelidia of staminodial origin, and the mentum of mixed sepaloid-petaloid-staminodial origin. *SEP* clade 1 copies are expressed in the larger callus of *P. equestris*, whereas *AP3* clade 1 and *AGL6* clade 1 copies are expressed in the pronounced mentum and long stelidia of *P. pulcherrima. AP3* clade 4, *PI*-, *AGL6* clade 2 and *PCF* clade 1 copies might have a balancing role in callus and gynostemium development. There appears to be a trade-off between *DIV* clade 2 expression with *SEP* clade 1 expression in the callus, on the one hand, and with *AP3* clade 1 and *AGL6* clade 1 expression in the stelidia and mentum on the other.

**Conclusions:**

We detected differential growth and expression of MADS box *AP3/PI*-like, *AGL*6-like and *SEP*-like, and MYB *DIV*-like gene copies in the callus, stelidia and mentum of two species of *Phalaenopsis,* of which these floral structures are very differently shaped and sized. Our study provides a first glimpse of the evolutionary developmental mechanisms driving adaptation of *Phalaenopsis* flowers to different pollinators by providing combined micro-morphological and molecular evidence for a possible sepaloid–petaloid–staminodial origin of the orchid mentum.

## Background

One of the key innovations in the evolution of flowering plants is the transfer of pollen by pollinators. This adaptation enabled plants to reproduce much more efficiently and played a major role in the diversification of the flowering plants [1]. In more basal flowering plant lineages, such as Magnoliales, pollinators carry pollen all over their body, resulting in a low pollination success. In more derived plant lineages such as orchids, very precise placement of pollen on very specific body parts of pollinators evolved, that ensured reproductive isolation and species diversification.

Apart from very precise pollen placement in orchids, deceptive pollination also evolved, in which pollinators are cheated when they visit flowers that offer no reward. Deceptive pollination promotes cross-fertilization by reducing the visitation period of pollinators and discouraging returns to a single flower or inflorescence, in this way preventing self-pollination. Seven mechanisms of deceptive pollination of orchids have been described so far: generalized food deception, Batesian floral mimicry, brood-site imitation, shelter imitation, pseudo-antagonism, rendezvous attraction and sexual deception [2]. The generalized food deception syndrome [3] is characterized by a model mimicking general floral signals of rewarding plants such as a similar inflorescence shape, floral color, scent, nectar guides, spurs and pollen-like papillae [4, 5]. These signals attract recently emerged, immigrant or exploratory pollinators [2]. The Batesian mimicry syndrome copies one particular rewarding model plant species growing nearby [6–8]. In the case of brood-site imitation, orchids deceive insects by mimicking oviposition substrates such as rotting fruit, dung, or fungi [9]. In the case of shelter imitation, flowers are shaped in the form of a tube [10] to provide shelter or a warm up place for insects in need of thermoregulation [11, 12]. The pseudo-antagonism syndrome relates to foraging behavior of kleptoparasitic pollinators by employing small floral hairs easily vibrating in a small breeze that are thought to mimic insect prey captured in spider webs [13, 14]. The first step in evolution towards pseudocopulation might be related with the rendezvous syndrome [15]. In the latter syndrome, flowers emit signals that attract pollinators of both sexes such as aggregation pheromones [16]. In the first syndrome, flowers mimic the shape and scent of female insects; this elicits male insect sexual behavior with the mimicking flower [17–19]. From the seven strategies described above, the most common one in orchids is general food deception. Around one-third of all pollination cases in orchids are categorized as general food deceptive [20]. Deceptive flowers look, feel and smell like flowers of nearby rewarding plants and only after visiting a cheating flower several times, inexperienced pollinators learn to distinguish a cheater from the model. This is sufficient to transfer pollen from one orchid to another and pass on this trait to future generations. Essential for the betrayal is that the deceptive flower manages to position the pollinator very accurately in front or below the stamen to remove pollen, and in a subsequent visit the stigma, to deposit pollen for cross-fertilization. Several floral organs and structures in different floral whorls play an important role in this process.

**Fig. 1:**
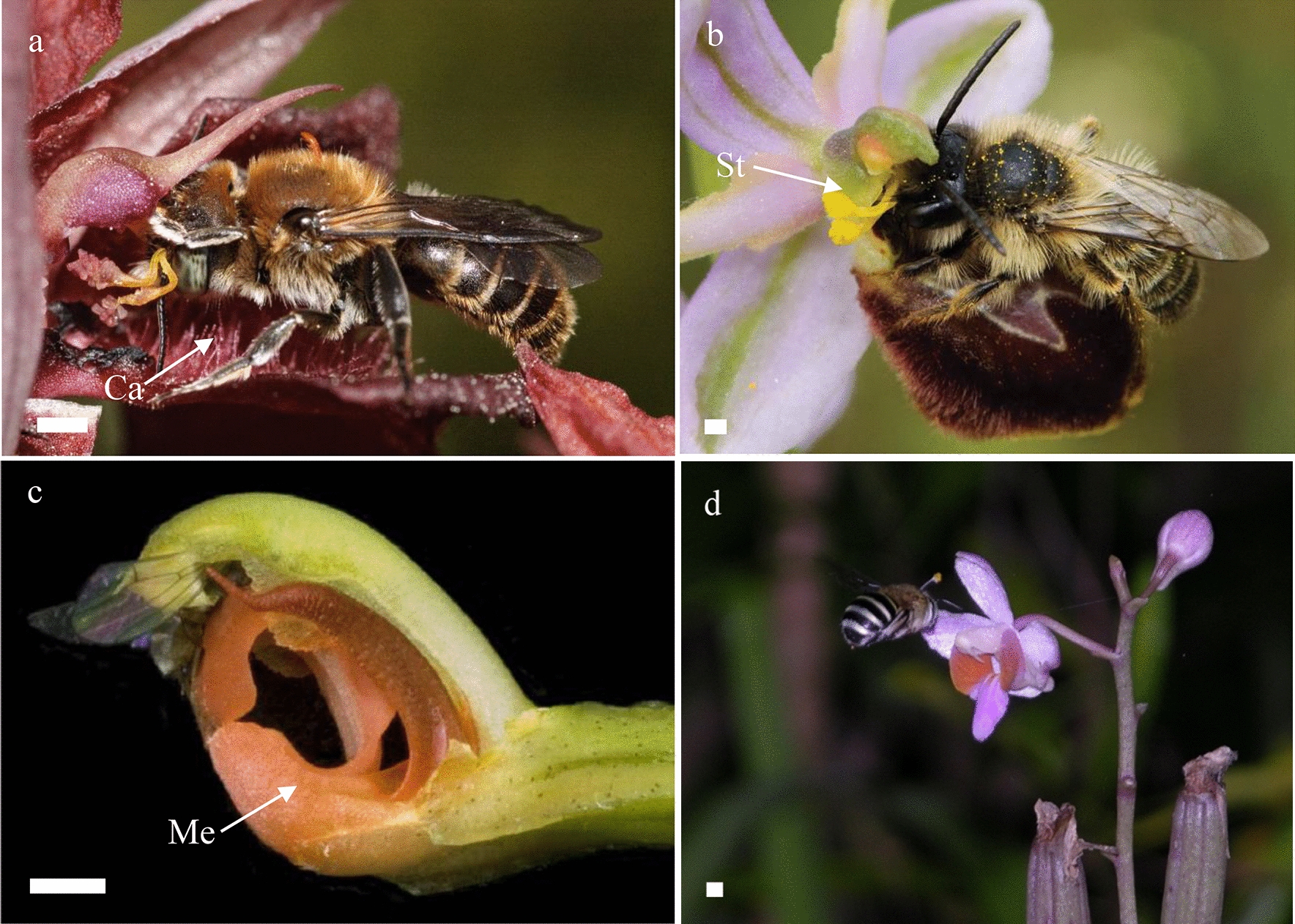
Variation in shape and size of floral callus, mentum, stelidia and corresponding orchid pollinators. **a** An *Hoplitis adunca* male bee is pollinating a *Serapias cordigera* flower in France, its front legs grab the callus on the labellum, while the pollinia are attached to its forehead; **b** an *Andrena* sp. male bee receives pollinia on its proboscis while pseudocopulating with a flower of *Ophrys splendida* in France; **c** a *Drosophila* sp. fly is trapped against the anther, while pollinating a flower of *Specklinia spectabilis* in Costa Rica; **d** an *Amegilla nigritar* bee bears a pollinarium on its head while approaching a flower of *Phalaenopsis pulcherrima* in China. *Ca* callus, *Me* mentum, *St* stelidia. Scale bars: 1 mm. Photographs by Jean Claessens (**a**, **b**), Adam Karremans (**c**) and Jin Xiaohua (**d**)

**Fig. 2:**
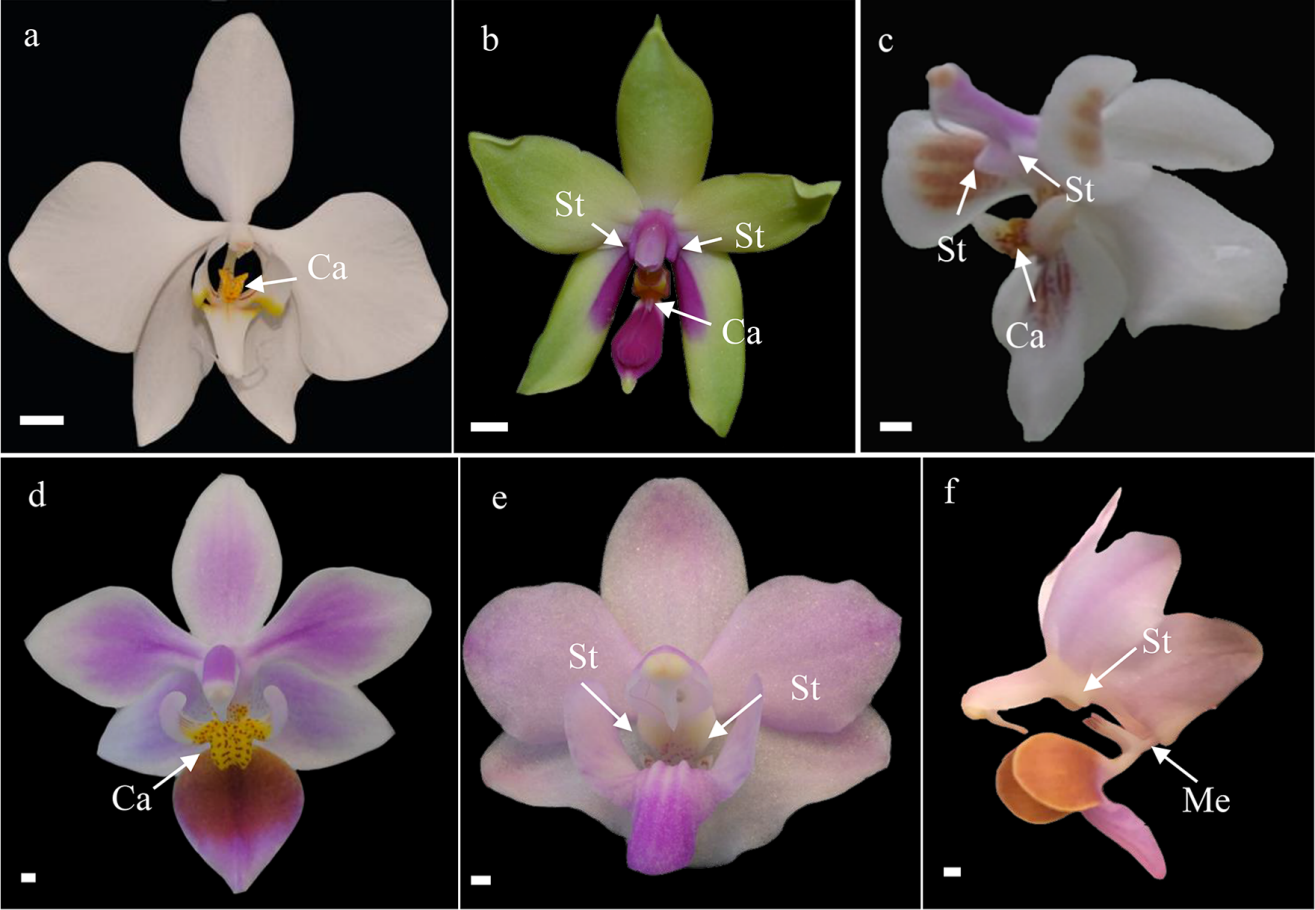
Variation in shape and size of floral callus, mentum and stelidia in the orchid genus *Phalaenopsis*. **a**. *Phalaenopsis amabilis*; **b**. *P. bellina;* **c**. *P. celebensis;* **d**. *P. equestris*; **e**. *P. pulcherima*; **f**. *P. pulcherrima*. Abbreviations: Ca = callus; Me = mentum; St = stelidia. Scale bar: 100 µm. Photographs by Rogier van Vugt (a) and Dewi Pramanik (b-f)

Orchid flowers consist of five whorls. The two outer whorls contain three sepals and three petals, of which the median petal is transformed into a labellum. There are two staminal whorls. The outer whorl consists of two staminodes and one functional stamen. The inner whorl is assumed to contain three staminodes but this is a long debated issue. In the center of the flower there is a fifth whorl that contains a gynoecium that is formed by three fused carpels and at least one stamen. The median petal of orchid flowers, the labellum, is often enlarged and ornamented with a wart-like structure, the callus. During landing on the orchid flower, the callus on the labellum provides a holdfast for the insect to grasp with its front legs [21] (Fig. 1a). The reproductive organs (style, stigma, stamen) have become fused and incorporated in a gynostemium [22, 23]. To prevent the insect from walking off the labellum, wing-shaped structures on both sides of the gynostemium, so-called stelidia [24], are vital as they keep the insect trapped and also position the head or other body parts of the animal correctly in front of the reproductive organs (Fig. 1b). Lastly, the so-called mentum, an outgrowth formed by the bases of the gynostemium and labellum and the lateral sides of the lateral sepals [25] can act as a hinge, slamming an insect that walks over the labellum against the upper part of the gynostemium, where the anther and stigma are positioned, to ensure that removal and/or deposition of the pollinia takes place at a very precise position on the pollinator and orchid flower (Fig. 1c). The size and shape of these three highly specialized floral structures became adapted to the bodies of specific pollinators during orchid evolution. This resulted in diversification of the shape and size of these structures. Such a diversification can for example be found in different species of the orchid genus *Phalaenopsis*. Examples of a large callus can be found in *Phalaenopsis amabilis* (Fig. 2a)*, P. celebensis* (Fig. 2c), and *P. equestris* (Fig. 2d), pronounced stelidia are present in *P. bellina* (Fig. 2b)*, P. celebensis* (Fig. 2c) and *P. pulcherrima* (Fig. 2f), whereas a large mentum is present in *P. pulcherrima* (Figs. 1d and 2f).

### Models of floral organ development in orchids

Multiple molecular studies show that the floral development of orchids involves interaction of different MADS box and other genes that duplicated early during orchid evolution [26–31]. The genes that are involved in floral development are interpreted with different models. The first model developed was the ABCDE model and this includes the following genes: MADS box A-class gene *APETALA1* (*AP1*), B-class genes *APETALA3* (*AP3*) and *PISTILLATA* (*PI*), C-class gene *AGAMOUS* (*AG*), D-class gene *SEEDSTICK* (*STK*) and E-class gene *SEPALLATA* (*SEP*) [32]. Combinations of multiple MADS box genes are required to form different floral organs in the petaloid monocots to which orchids belong: class A + B + E genes specify sepals and petals, B + C + E determine stamens, C + E specify carpels, and D + E are necessary for ovule development [33, 34]. Since this model could not entirely explain the shape of orchid flowers, it had to be expanded. First of all, petaloid monocots do not possess AP1 orthologues and *FRUITFULL* (*FUL*)-like genes are the closest homologues available [35]. The development of the orchid labellum cannot not be explained with the ABCDE model, so the Homeotic Orchid Tepal (HOT) model was developed [36], followed by the Perianth code [29] and the Oncidiinae model [31]. According to the HOT model, four *AP3* gene copies (*AP3*-*1, AP3*-*2, AP3*-*3*, and *AP3*-*4*) and *PI*, in combination with other classes MADS-box genes, regulate the flower identity program, resulting in distinct sepals, petals and a labellum (median petal) in orchids [36]. The Perianth code model explains the ratio of two complexes of the MADS-box proteins *AP3/AGL6/PI*, called SP and L, respectively, that play a pivotal role in sepal-petal development and also determine the identity of the labellum. When the complexes are skewed towards the L-complex, the labellum is large; when they are skewed towards the SP complex, the labellum is small or absent [29]. The Oncidiinae model summarizes differential expression of selected MADS-box genes in the perianth of *Oncidium* Gower Ramsey and *Erycina pusilla*. Clade 1 *AP3*-like *OMADS5* and *EpMADS15*, and clade 1 *AGL6*-like genes *OMADS7* and *EpMADS3* are expressed in the sepals and petals of both species. Clade 2 *AP3*- like *OMADS3* is expressed in the entire perianth of *O.* Gower Ramsey, while its ortholog, *EpMADS14*, is only expressed in the lateral sepals of *E. pusilla*. Moreover, clade 3 *AGL6*-like gene *EpMADS4* is solely expressed in the lateral sepals of *E. pusilla,* causing the median sepal to look different from the lateral ones in this species [31].

Other transcription factors that play an important role in shaping zygomorphy of flowers are RADIALIS (RAD), DIVARICATA (DIV) and DRIF (Divaricata Radialis interacting factor) that belong to the *MYB* gene family. They interact with each other and with TCP family genes to regulate floral dorsiventral asymmetry. In *Antirrhinum majus, DIV* promotes the ventral identity of petals [37], and *RAD* promotes the dorsal identity by binding to *DRIF* and preventing the formation of a *DIV/DRIF* complex. *RAD* and *DIV* interact with *DRIF* in the regulation of floral symmetry. In the dorsal region, *CYC/DICH* genes interact with the promotor and intron of the *RAD* gene to activate its expression [38]. RAD proteins bind to *DRIF* I, preventing the binding of *DIV* with *DRIF,* and ensure that a *DIV/DRIF* complex cannot be formed. In the ventral region, the absence of *RAD* allows *DIV* to interact with *DRIF*, and this allows the formation of a *DIV/DRIF* complex that promotes the expression of genes that are specific to the ventral region of flowers [39]. Recently, it was discovered that MYB transcription factors play a role in shaping zygomorphy of the orchid flower as well [40, 41]. It was detected that the MYB transcription factors DIV, RAD and DRIF can form a regulatory module that enriches the orchid developmental code [42, 43].

### MADS-box genes drive the development of the orchid callus, stelidia and mentum

So far, the expression of genes related to the development of a callus on the orchid labellum was only reported for *E. pusilla.* Dirks-Mulder et al. [31] found a mixed petaloid-staminodial origin for this particular organ based on combined micro-CT evidence and expression of MADS box A (*FUL*-like), B (*AP3, PI*), E (*SEP*) and *AGL6*-*2* genes. These results are in line with the ABCDE model, that predicts MADS box B-class genes in organs derived from petals, and the Perianth code model, which predicts expression of *AGL6*-*2* genes in a labellum-derived organ.

MADS-box genes involved in the development of the gynostemium are *AGAMOUS* and *SEEDSTICK* [44–49]. Gene expression in the stelidia was so far only studied in *E. pusilla* [31]. In this study, a staminodial origin was found based on combined micro-CT evidence and expression of MADS box A (*FUL*-like), B (*AP3, PI*), C (*AG*), D (*STK*), E (*SEP*) and *AGL6*-*3* genes. These results are in line with the ABCDE model, that predicts expression of MADS box B and C-class genes in organs derived from stamens. To the best of our knowledge, no gene expression analyses of the orchid mentum have been reported yet.

To understand more about the evolution and development of the orchid callus, stelidia and mentum, and the MADS-box and MYB transcription factors expressed in these structures, two different *Phalaenopsis* species, *P. equestris,* and *P. pulcherrima* were studied with a combination of micro-morphological and molecular techniques. A phylogenetic analysis of *Phalaenopsis* based on the nuclear internal transcribed spacers (nrITS) and plastid DNA showed that both species are a member of subgenus *Phalaenopsis. Phalaenopsis equestris* is part of section *Stauroglottis*, whereas *P. pulcherrima* is a member of section *Esmeralda* [50]. These species differ in terms of floral morphology as the first has a large callus, short stelidia and no mentum, whereas the second has a small callus, long stelidia and a pronounced mentum. Species of *Phalaenopsis* with a large callus, short stelidia and an inconspicuous mentum are pollinated by large pollinators such as *Xylocopa* bees [51, 52] whereas species with a small callus, long stelidia and a conspicuous mentum, like *P. pulcherrima*, are pollinated by smaller pollinators such as *Amegilla* bees (Fig. 1d) [53]. The expectation was that the differences in floral morphology of *P. equestris* and *P. pulcherrima* are correlated with differential expression of a selection of MADS box genes earlier found to be expressed in the callus and stelidia of flower of *E. pusilla* and other transcription factors. *Phalaenopsis* and *Erycina* belong to the Aeridinae and Oncidiinae tribes, respectively, that are both members of the so-called ‘advanced’ Epidendroideae according to a phylogenetic analyses based on the nuclear internal transcribed spacers (nrITS), nuclear xanthine de-hydrogenase (*XDH*), mitochondrial *nad1* and plastid DNA [54]. Questions that we aimed to answer were (i) is the callus in *Phalaenopsis* flowers of mixed petaloid-staminodial origin, (ii) are the stelidia of *Phalaenopsis* flowers derived from staminodes, (iii) what is the evolutionary origin of the mentum in *Phalaenopsis,* and (iv) can differences in the size and shape of the callus, mentum and stelidia of *P. equestris* and *P. pulcherrima* be explained by differential expression of MADS box and/or MYB transcription factors?

## Materials and methods

### Plant material

**Fig. 3:**
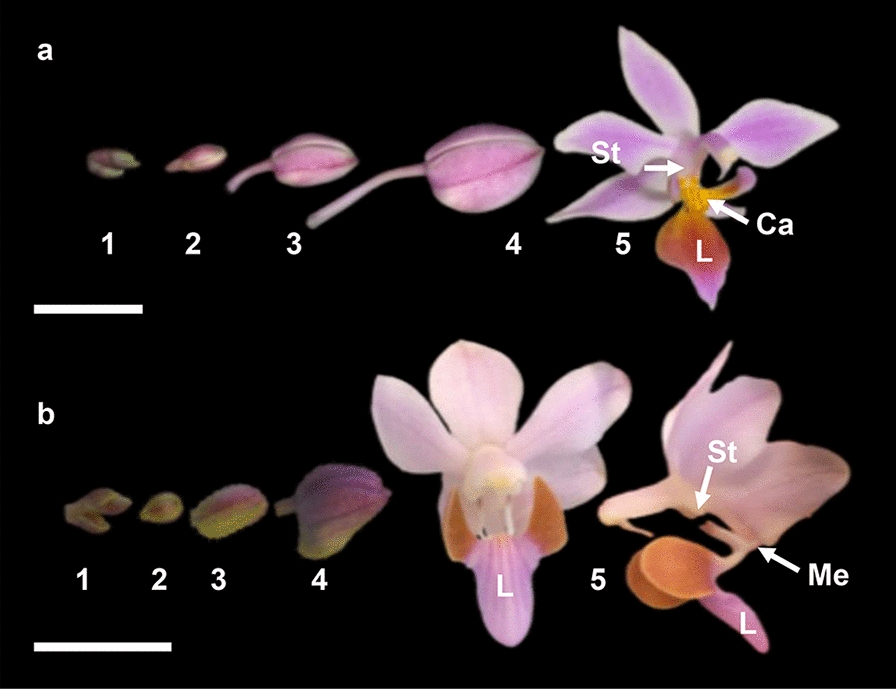
Different floral developmental stages of *Phalaenopsis equestris* and *P. pulcherrima.* **a**. *P. equestris;* **b**. *P. pulcherrima.* Abbreviations: Ca = callus; L = labellum; Me = mentum; St = stelidia. Scale bar: 1 cm. Photographs by Dewi Pramanik

A total of three *P. equestris* and three *P. pulcherrima* plants, obtained from commercial growers, were reared in the tropical greenhouses of the Hortus botanicus of Leiden University. Based on the size of the floral buds, five different developmental stages were defined (Fig. 3). Floral samples were freshly collected and stored at − 80 °C for molecular analysis or in standard formalin-aceto-alcohol (FAA: absolute ethanol, 90%; glacial acetic acid, 5%; formalin acetic acid, 5%) for imaging. Floral organs for SEM imaging and fully open flowers for micro 3-D CT scanning were immersed in FAA solution for 1 h under vacuum pressure at room temperature, then left for a minimum of 7 days under room temperature.

### Scanning electronic microscopy (SEM)

Spirit samples of floral organs and structures preserved in a FAA solution were first transferred to 70% ethanol solutions and then further dissected under light microscope (Leitz). Subsequently the samples were washed in 96% ethanol (two times, 20 min) and in 96% acetone (two times, 30 min). To dry the samples and remove any presence of water, a critical point dryer (Leica EM CPD300) was used. The samples were mounted on aluminum stubs of 12.2 × 10 mm diameter (Ted Pella, Inc) using a conductive carbon cement (Structure Probe Inc, United States) or sticker glue (Carbon adhesive tabs, EMS). To inhibit charging, reduce thermal damage, and improve secondary electron emission, the samples were coated with a Platina-Palladium layer of 20 nm, by using a sputter coater (Quorum Q150TS). Sample observations were obtained using a JSM-7600F SEM field emission scanning electron microscope (JEOL).

### Micro-3-D computed tomography scanning

Spirit samples of mature flowers stored in FAA were treated with 0.1% Phosphotungstic acid (PTA) in 70% ethanol over a timespan of five days and daily refreshed. Prior to scanning, the samples were washed in 70% ethanol two times for 30 min each, then embedded in 1.5% low melting point agarose (Promega). The scans were performed on a Zeiss Xradia 510 Versa 3D X-ray with a Sealed transmission of 30–160 kV, with a maximum of 10 W X-ray source. Scanning of the flower was performed using the following settings: lens magnitude 0.5x; acceleration voltage/power 80 kV/7 W; exposure time 21 s; projections 1400. The total exposure time was approximately 9 h and 17 min.

### Vascular bundles reconstruction and serial layer sectioning

Image stacking and vascular bundle reconstruction was performed in Amira-Avizo 9.5 (Thermo Fisher Scientific). With this software, 3-D structures were made through voxels (3-D pixels). Vascular bundle coloration was produced by creating a script pipeline on the project panel and coloring the bundles in the segmentation editor. Through 2-D segmentation of the images, and electronic volume removal of the lobes, the vascular bundles in the callus, mentum and stelidia could be studied. To better understand organ development, serial layer sectioning was produced from processed 3-D CT scan images. The layer sectioning was made using the script of the bounding box and color wash.

### RNA extraction

Floral buds were collected from stage 1, whereas the callus and gynostemium (including mentum and stelidia, but excluding the anther cap with pollinia) were dissected and collected from stage 5 (Fig. 3). A maximum of 100 mg of each sample was transferred into sterile 2.2-mL micro-centrifuge tubes, together with a 7-mm glass bead (Assistent), and stored at − 80 °C. The frozen samples were grinded by using a TissueLyser II (QIAGEN). RNA was extracted by using a RNeasy Plant Mini Kit (QIAGEN), under RNase-free conditions. DNase I Amp Grade (Invitrogen 1U/μl) was applied to the extracted RNA to digest single- and double-stranded DNA. To assess the quantity and quality of the samples for cDNA synthesis, they were measured with a nanodrop (NanoDrop 2000c, ThermoFisher), whereas for sequencing, they were measured by Agilent 2100 Bioanalyzer Systems (Agilent Technologies). The quantity of the RNA samples analyzed was at least 50 ng/μL RNA in 50 μL volume, the quality as assessed by the RNA integrity Number (RIN) was at least 7. All RNA samples with a RIN < 7 were discarded. Samples used for further downstream experiments were stored at − 80 °C.

### Transcriptomic and bioinformatic analyses

RNA samples isolated from three biological replicates of early floral buds, mature callus and gynostemium of *P. equestris* and *P. pulcherrima* were used for sequencing, and they were processed separately per organ. These samples were sequenced by the Beijing Genome Institute (BGI) using de novo sequencing with an Illumina HiSeq PE150. BGI cleaned the generated reads and uploaded these to their cloud service. The cleaned reads were downloaded and further analyzed with an in-house designed bioinformatics pipeline [55] using the Naturalis OpenStack server, which was accessed via PuTTY, a free SSH and telnet client (https://www.putty.org/). An in-house designed bioinformatics pipeline was used for quality control, assembly, annotation and differential expression analysis. First, the reads were checked for quality with FastQC v0.10.1 [56]. Reads with low quality were trimmed or removed with Trimmomatic v0.32 [57]. With the cleaned reads a de novo assembly was made using Trinity v2.5.1 [58]. Trinity used Bowtie2 v2-2.3.3.1 [59] for aligning the reads. The output of Trinity was clustered to remove redundant data and reduce the size of the transcriptomes using CDHIT-EST [60]. Longer consensus transcripts called ‘unigenes’ resulting from the assembly step were subsequently annotated with gene names using a dedicated local orchid gene database that was made by filtering orchid sequences from the NCBI GenBank Nucleotide (nt) and Non-Redundant (nr) protein databases. The reads were aligned to these unigenes, which enabled the next step: creating a count table with expression values. This count table was based on Trinity identifiers, which were used to link the unigenes to the annotations.

### Differential gene expression analysis

To visualize differential gene expression, a count table with expression values was used to produce heatmaps in RStudio version 1.1.463. The heatmaps were generated from the raw number of times a specific read in the transcriptomes, generated from a specific organ at a specific time, could be mapped against a reference gene. If so, this was counted as a hit. In a separately generated ‘Color Key and Histogram’, the number of counts runs from 0 to 14 with 0 being the lowest up to 12 being the highest. Color codes were given based on the number of counts found in the different samples analyzed divided by the total number of counts. The script was run separately for each species. Genes with too low expression (< 100 reads) were removed from the dataset. We also carried out differential expression analyses by using DESeq in RStudio [61], and these results are presented in Additional file 1: Table S4, depicting the Log fold change of expression of all developmental genes analyzed. An analysis was carried out using Wald statistics to assess the significance of the results. The results presented in Additional file 1: Table S4 and Table S5 are congruent with the relative expression levels shown in the heatmaps depicted in Figs. 7 and 8.

### cDNA synthesis

cDNA was reverse-transcribed through reverse transcriptase (RT) by using the iScript cDNA Synthesis Kit (Bio-RAD). The reaction mix (20 µL) consisted of 5 × iscript reaction mix (4 µL), iscript reverse transcriptase (1 μL), 1 μg RNA template and water for the rest of the volume. The cDNA was synthesized under the following conditions: priming of the RT at 25 °C for 5 min; reverse transcription at 46 °C for 20 min; RT inactivation at 95 °C for 1 min.

### Primer design

Nucleotide sequences of MADS-box A, B, C, D and E class genes of *Phalaenopsis* and control genes *PeACT* and *PpACT* (*Actin*) were generated from reads obtained from the transcriptomes. Specific primers were designed in Geneious version 8.1.8 (http://www.geneious.com) (Additional file 1: Table S1). For screening of binding specificity, all primer pairs were blasted against the Orchidstra database and further tested using PCR and gel electrophoresis.

### Semi-quantitative reverse transcriptase PCR (RT-PCR)

A semi-quantitative RT-PCR was used to quantify the expression of selected MADS box genes. Total cDNA from three biological replications of early floral buds, mature callus and gynostemium of *P. equestris* and early floral buds, mature callus and mix of gynostemium, stelidia and mentum of *P. pulcherrima* was analyzed in triplo for each primer set. *PeACT* and *PpACT* were used as positive control, while the non-template control (reaction without cDNA template) was assigned as negative control. Each amplicon group contained the tested samples, a positive control, and a non-template control. The amplification reaction was prepared by using 90 ng cDNA template. The reaction mix contained 10 × CoralLoad Buffer (Qiagen), 25 mM MgCl_2_ (Qiagen), 100 mM Bovine Serum Albumin, Acetylated-BSA (Promega), 1.25x DMSO (Qiagen), 5x Q-Solution (Qiagen), 0.2 μM of forward and reverse primer (IDT), 2.5 mM dNTPs (Qiagen), 1.25 units/50 µl DNA *Taq* Polymerase (Qiagen), and milliQ water (Ultrapure) was used to reach the final volume of 25 µl. The amplification protocol started with a polymerase activation and initial denaturation at 94 °C for 5 min; continued with 35 cycles of denaturation at 94 °C for 30 s; then an annealing step at 56 °C for 30 s; an extension step at 72 °C for 1 min 40 s; and a final extension at 72 °C for 7 min. PCR products were run on a 1.0% agarose gel (DNAase/RNAase-free, Bioline) in 0.5× Ultrapure Tris–Borate–EDTA (TBE) buffer (ThermoScientific) for 30 min at 100 V. The gels were stained with ethidium bromide (Gibco-BRL) for 30 min and digitally photographed using an Ultima 10si gel doc system (ISOGEN, Life Science).

### Phylogenetic analysis

A multiple sequence alignment was performed by using the ClustalW alignment tool within Geneious version 8.1.8 based on translated nucleotides. The nucleotide sequences of MADS box genes of *P. equestris* and *P. pulcherrima* from transcriptome analysis were first translated to amino acids in the correct translation frame (Additional file 1: Table S2). The reference protein sequences of floral development genes were downloaded from databases (Additional file 1: Table S3). Additional references of *Orchis italica* and *Apostasia shenzenica* MADS box genes sequences were obtained from Valoroso et al. [40] and Zhang et al. [62], respectively. The created alignment was trimmed down to the most conserved regions (protein domains and amino acid motifs) to make sure that all sequences had the same length. Regions that did not align were removed prior to further analysis and the presence/absence of shared indels was coded with an extra 1 or 0. Phylogenetic trees were generated with the Geneious Tree Builder plug-in using the Maximum Likelihood (ML) method with gymnosperm gene lineages as outgroup based on Pabón-Mora et al. [63]. A bootstrapping of 100 times was carried out, of which the resulting support values are shown as numbers above the branches.

## Results

### Floral ontogeny and micromorphology

**Fig. 4:**
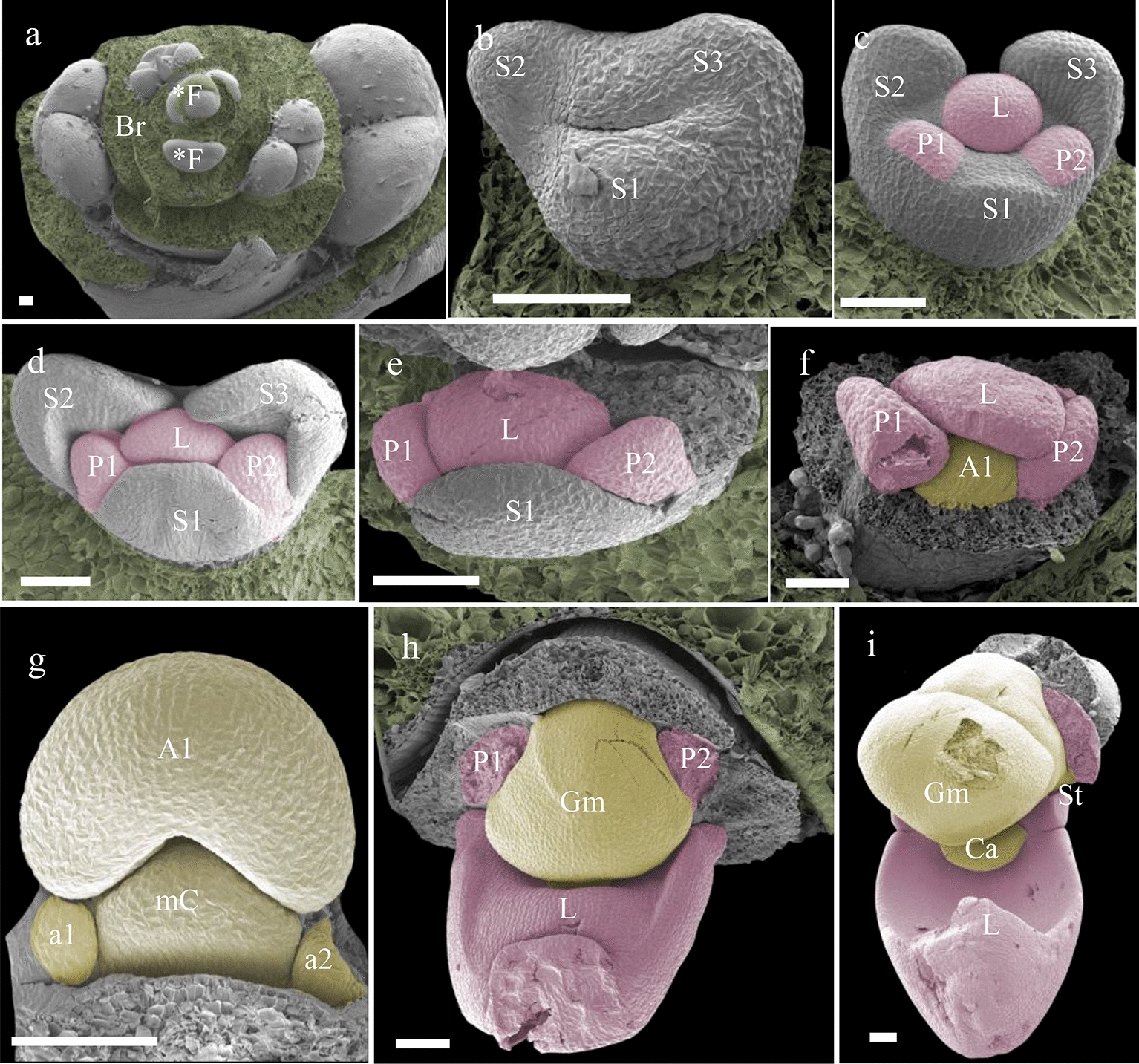
Floral ontogeny of *Phalaenopsis equestris*. **a** Undifferentiated oval-shaped early stage primordia covered by bracts, ×50 magnitude; **b** The transversally stretched bulges of early floral development with the initiation of sepals, ×350 magnitude; **c–e** further differentiation and development of sepals, petals and labellum of a single flower, ×200**–**×150 magnification; **e–f** an almost fully developed labellum and initiation of anther, ×150**–**×130 magnification; **g** further differentiation of median carpel, anther and staminodes, ×100 magnitude; **h** almost full-grown gynostemium, ×70 magnification; **i** halfway developed callus on the labellum, ×45 magnification. Scale bar: 100 µm. *F primordial floral bud; *A1* anther, *a1****–****a2* staminodes, *Br* bracts, *Ca* callus, *Gm* gynostemium, *L* labellum, *mC* median carpel, *P1****–****P2* abaxial petals, *S1****–****S2* abaxial sepals, *S3* median sepal, *St* stelidia. Scale bar: 100 µm. Photographs by Dewi Pramanik

**Fig. 5:**
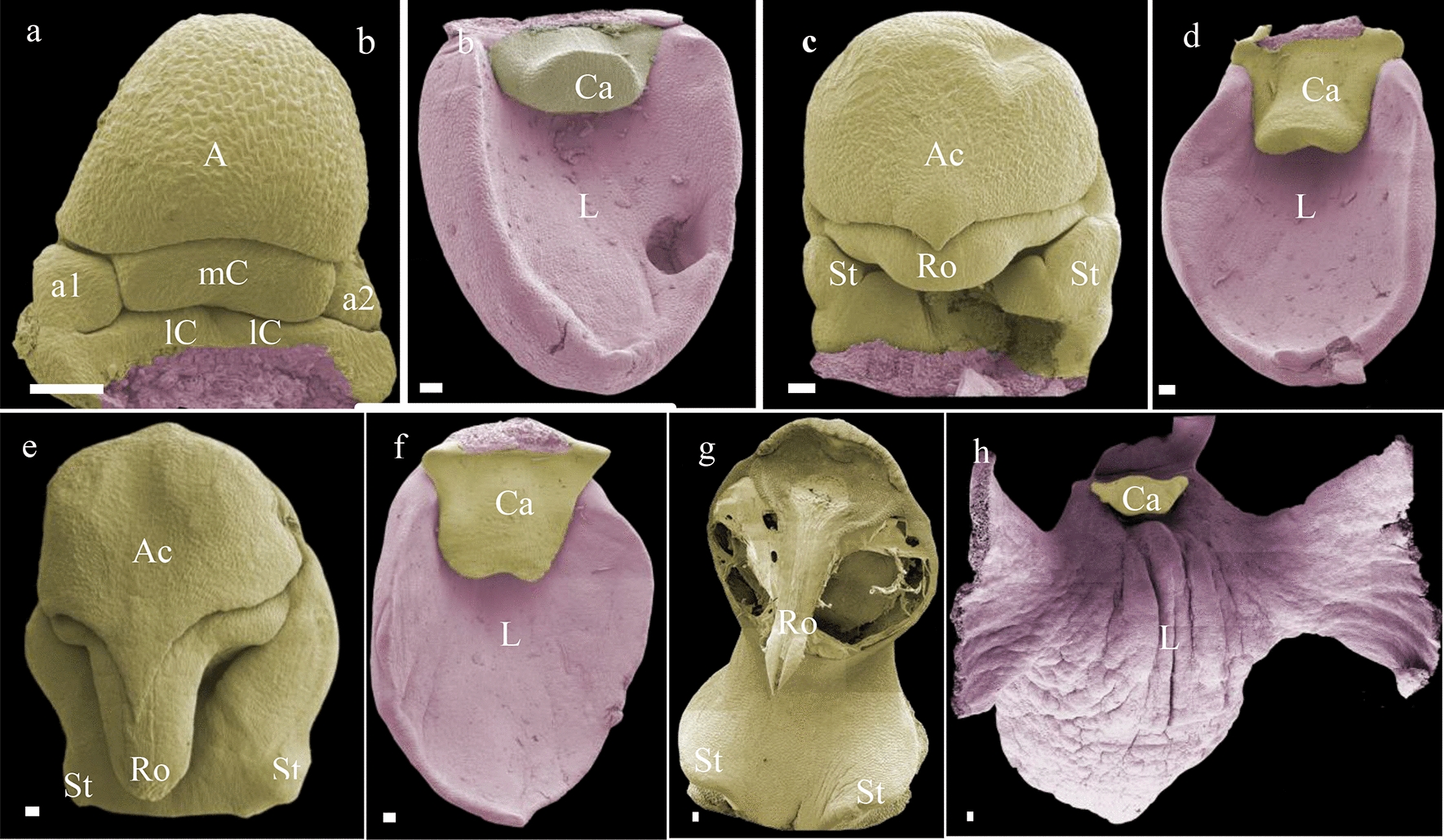
Floral ontogeny of *Phalaenopsis pulcherrima*. **a** Early stage gynostemium in which the staminodes are still visible as separate organs; **b** early formation of the callus on the labellum; **c**, **d** late stage floral bud in which the size of the anther cap, stelidia, rostellum, callus and labellum further increases; **e** almost fully developed apical part of the gynostemium, the rostellum pokes out from underneath the anther cap and the stelidia are almost fully merged with the gynostemium; **f** early stage labellum and almost fully developed callus; **g** late stage apical part of the gynostemium, from which the anther cap and pollinia were removed, showing the fully developed rostellum; h. fully developed labellum and callus. Images were made under various magnifications ranging from ×37 to ×130 magnification. *A1* anther, *a1–a2* staminodes, *Ac* anther cap, *Ca* callus, *L* labellum, *lC* lateral carpel, *mC* median carpel, *St* stelidia, *Ro* rostellum. Scale bar: 100 µm. Photographs by Dewi Pramanik

The floral development of *Phalaenopsis* can be divided into five main phases (Fig. 3). Early ontogeny starts with floral primordial initiation (stage 1). In this stage, the floral primordia (*F) are protected by specialized bracts (Br). When these bracts are removed, the primordia become visible as oval-shaped structures (Fig. 4a). The oval-shaped primordia become transversally stretched bulges after early development of the sepals (S1–S3) (Fig. 4b). The development of the floral organ is followed by the abaxial petals (P1–P2), immediately followed by the labellum (L) (Fig. 4c-e). Subsequently, anther (A) development starts with the formation of a bulge-like structure on the apex in the shape of an abaxial ridge (Fig. 4f). This ridge differentiates into anther, median carpel, and staminodes (Fig. 4 g), and in later development, these structures gradually fuse with the gynostemium (Gm) (Fig. 4 h). In late stage 1, the anther, median carpel, and staminodes are fully fused in the gynostemium and a callus on the hypochile starts to develop (Fig. 4i). The stelidia appear at each side of the gynostemium at the beginning of developmental stage 2, from which they further elongate. This part of the floral ontogeny was largely congruent between *P. equestris* (Fig. 4) and *P. pulcherrima* (data not shown). Compared to *P. equestris*, the callus in *P. pulcherrima* remained smaller, but the stelidia elongated much more during developmental phases 3-5 (Fig. 5a–e). The mentum of *P. pulcherrima* flowers starts to form in developmental stage 4 (Fig. 3b).

**Fig. 6:**
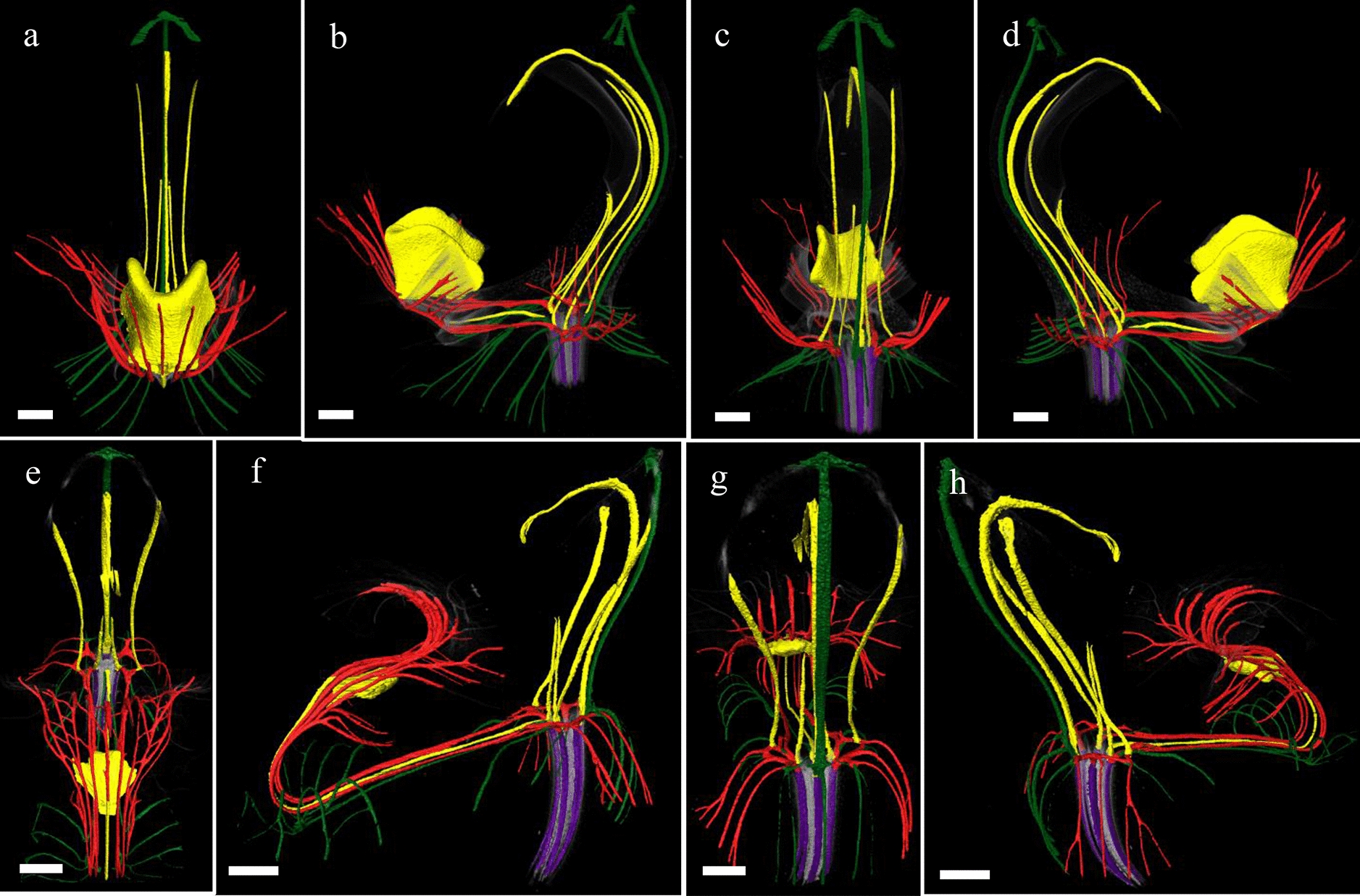
Reconstruction of vascular bundle patterns in a mature *Phalaenopsis* flower based on μCT scanning. **a–d** *P. equestris*; **e–h**. *P. pulcherrima*. **a** Frontal view; **b** successive clockwise turn of 45 degrees; **c** successive clockwise turn of 90 degrees; **d** successive clockwise turn of 135 degrees; **e** frontal view; **f** successive clockwise turn of 45 degrees; **g** successive clockwise turn of 90 degrees; **h**. successive clockwise turn of 135 degrees. Color codes: purple = vascular bundles in ovary; yellow = vascular bundles in stamen and staminodes; green = vascular bundles in sepals; red = vascular bundles in labellum and petals. Scale bar: 100 μm

To study the anatomy of mature flowers of *P. equestris* and *P. pulcherrima*, and verify the results from the SEM analyses, micro 3-D CT scans were made from mature stage 5 flowers of *P. equestris* (Fig. 6a–d; Additional file 2: Movie S1) and *P. pulcherrima* (Fig. 6e–h; Additional file 2: Movie S2). Six vascular bundles could be visualized in the inferior ovary, indicated in purple (Fig. 6b–h). Three of these bundles, indicated in green, run to the median and abaxial sepals (Fig. 6b–h). Three main groups of bundles, indicated in red, were found to feed the petals, including the labellum, in which they further split up (Fig. 6a–h). Four vascular bundles, indicated in yellow, could be detected, running towards the fertile stamen and staminodes. One of these bundles runs to the fertile stamen (Fig. 6b–d, f–h); two other bundles, originating from two pairs each, run up into the stelidia (Fig. 6a–h) and the remaining bundle runs all the way up into the callus on the labellum (Fig. 6a–h). The mentum is vascularized by bundles indicated in green (Fig. 6 g–h), red (Fig. 6e–h), and yellow (Fig. 6e–h). When following all vascular bundles downwards, they connect in a plexus situated on top of the inferior ovary, where they connect with the remainder of the vascular system of the flower.

### Duplications and expression of selected developmental genes in early floral buds and mature floral structures

Phylogenetic analyses recovered two clades of MADS box *FUL*-like genes, four clades of *AP3* genes, one clade of *PI* genes, three clades of *AG* genes, three clades of *STK* genes, four clades of *SEP* genes and three clades of *ALG6*-genes. Of the MYB transcription factors, we found three clades of *DIV* genes, one clade of *RAD* genes and three clades of *DRIF* genes. Of the TCP transcription factors, we found two clades of *PCF* genes, two clades of *CIN* genes and three clades of *CYC/TB1* genes (Additional file 3: Figs. S1–S11).

**Fig. 7:**
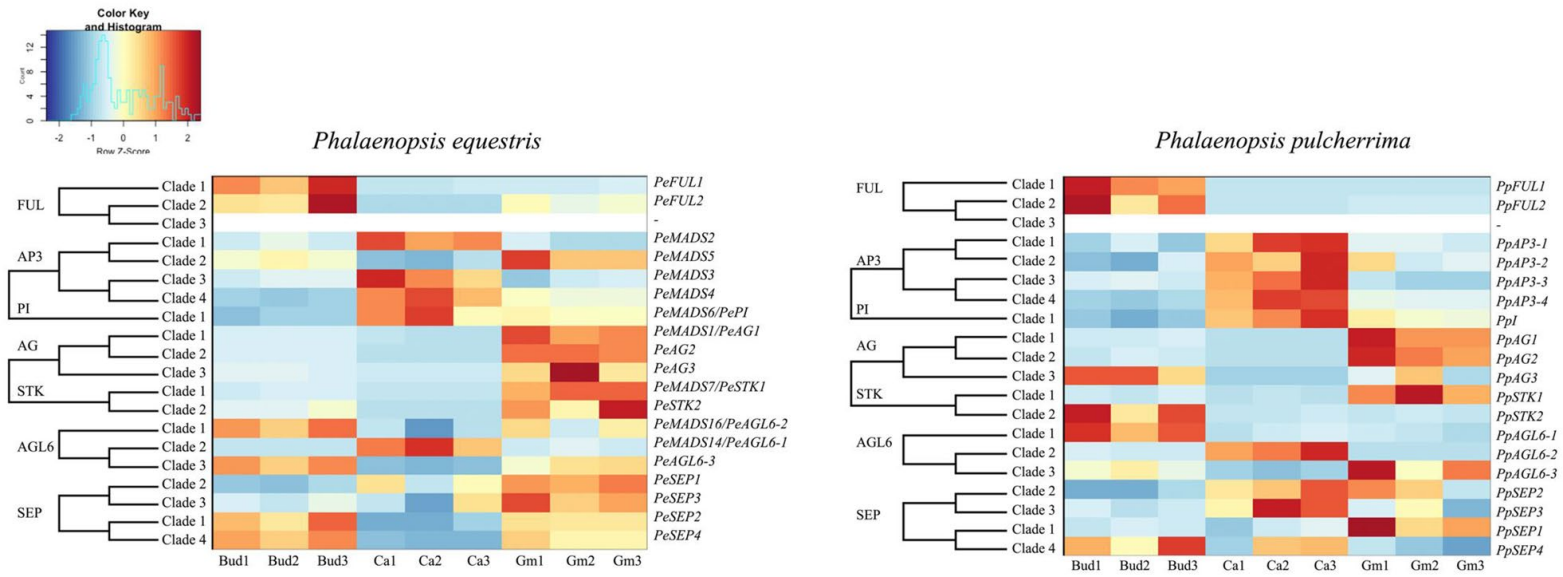
Heat map representation of number of counts of MADS-box gene expression in transcriptomes of early floral bud, mature callus and gynostemium of *P. equestris* and *P. pulcherrima.* Each sample had three biological replications. The scale of the heat map is based on the number of counts obtained from the transcriptome data. *Bud* early stage floral bud, *Ca* mature callus, *Gm* mature gynostemium

The transcriptome analysis showed that the different gene copies investigated were not expressed in all floral structures studied and the level of expression also varied. Below, we summarize the patterns found for the MADS box, MYB and TCP transcription factors. Of the MADS-box genes, the A-class genes were found to have a higher number of counts in early than late floral bud development. B-class genes were mostly expressed in the mature callus. C and D-class genes were highly expressed in the mature gynostemium. The *AGL6*-like and E class genes had variable differential expression patterns. In *P. equestris* these genes were predominantly expressed in the early floral bud and mature gynostemium, while in *P. pulcherrima,* these genes were mostly expressed in the mature callus and gynostemium (Fig. 7).

**Fig. 8:**
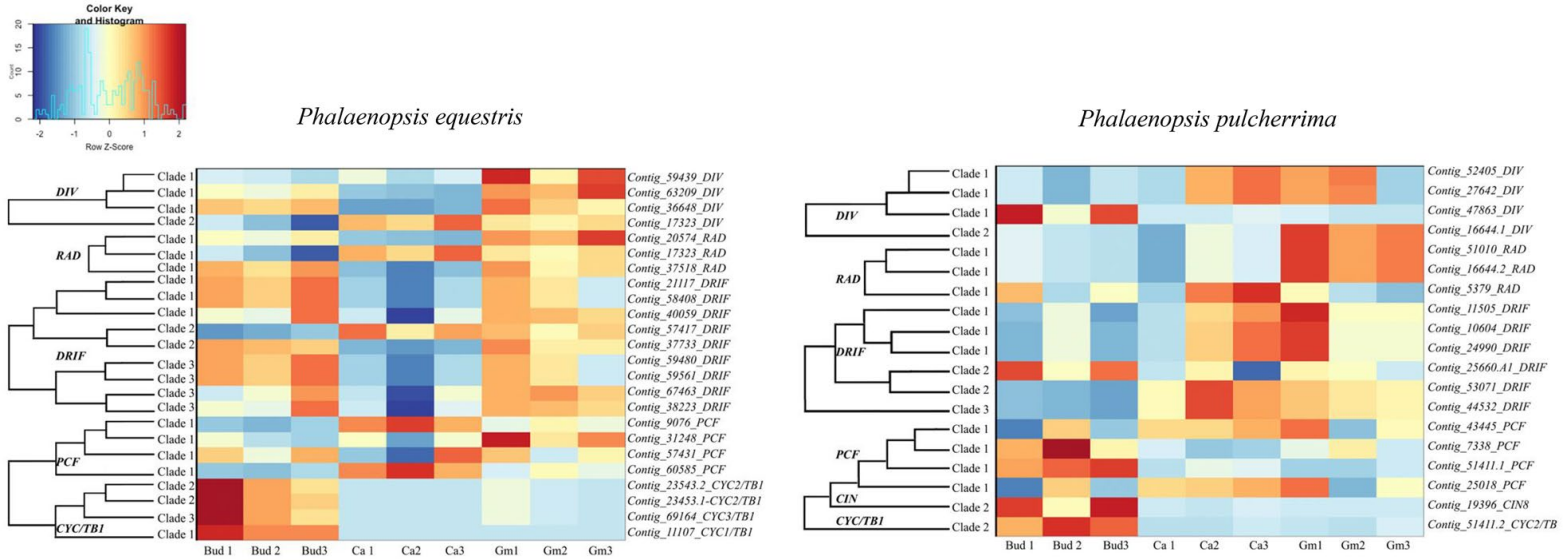
Heat map representation of number of counts of *MYB* and *TCP* gene expression in transcriptomes of early floral bud, mature callus and gynostemium of *P. equestris* and *P. pulcherrima.* Each sample has three biological replications. The scale of the heat map is based on the number of counts obtained from the transcriptome data. *Bud* early stage floral bud, *Ca* mature callus, *Gm* mature gynostemium

**Fig. 9:**
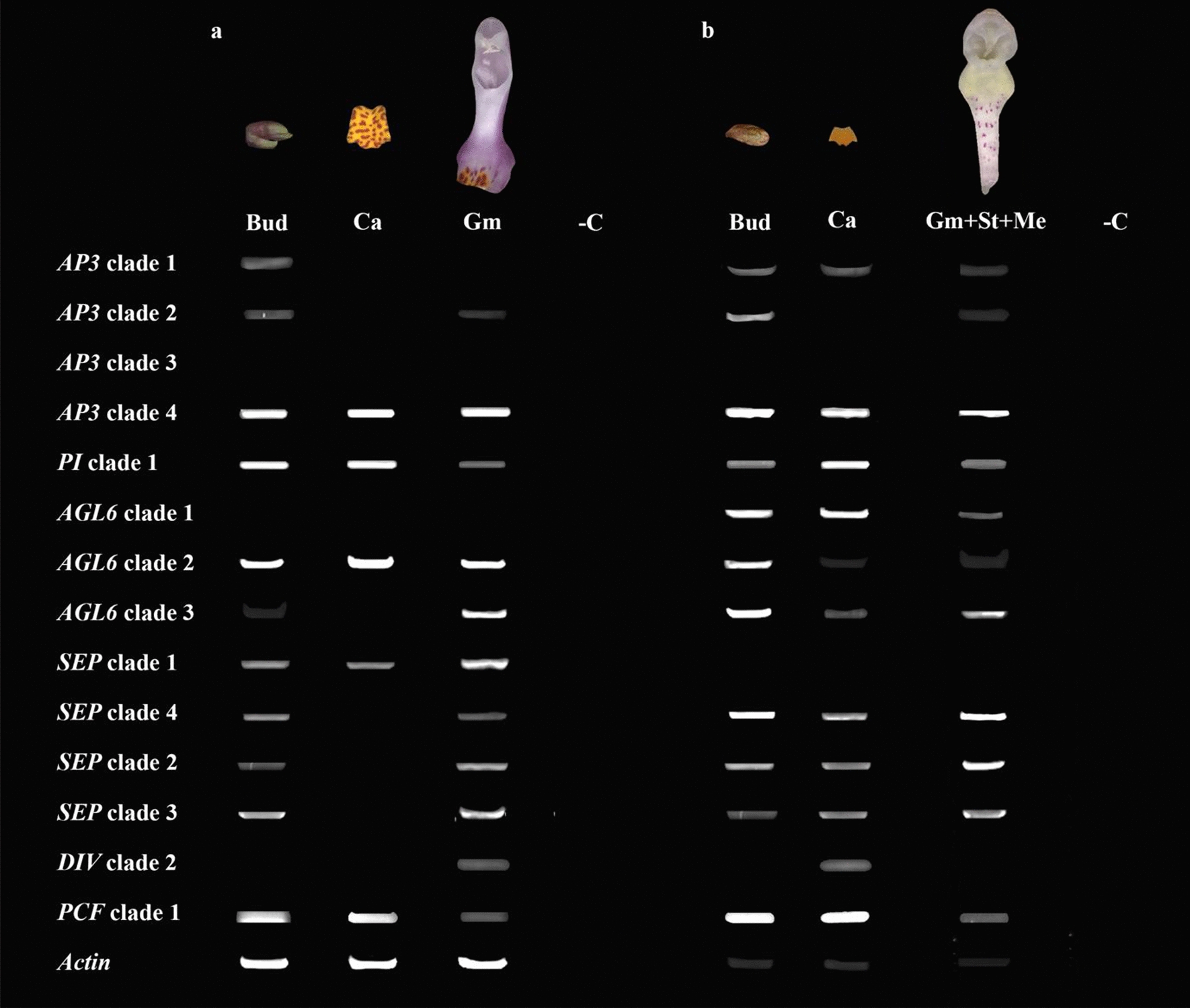
MADS-box, MYB and TCP genes expression in floral organs of *P. equestris* and *P. pulcherrima.* **a** Expression of isolated *AP3*, *PI*, *SEP*, *AGL6, DIV* and *PCF* genes in dissected floral organs of *P. equestris*; **b** expression of isolated *AP3*, *PI*, *SEP*, *AGL6, DIV* and *PCF* genes in dissected floral organs of *P. pulcherrima. Bud* floral bud stage 1, *Ca* callus, *Gm* gynostemium, *Gm + St + Me* gynostemium including stelidia and mentum, *–C* PCR amplification reaction without template (NTC)

*DIV* and *RAD* genes in both *Phalaenopsis* species had less expression in the early floral bud but more in the mature callus and gynostemium. The expression of *DIV* clade 2 was further analyzed using semi-quantitative RT-PCR. The *DIV* clade 2 gene was found to be highly expressed in the gynostemium of *P. equestris* and in the callus of *P. pulcherrima.* Similar to the *AGL6* and E-class genes*, DRIF* genes were highly expressed in the early floral bud and mature gynostemium of *P. equestris.* In *P. pulcherrima,* most *DRIF* copies were highly expressed in the mature callus and gynostemium. Most *TCP* genes were mainly expressed in the early floral bud, except for two *PCF*-like gene copies, that were expressed in the mature callus and gynostemium of both species of *Phalaenopsis* investigated (Fig. 8). RT-PCR showed that the *PCF* clade 1 lineage was highly expressed in all tissue types analyzed in both *Phalaenopsis* species (Fig. 9). To further understand the molecular basis of the development of the callus, stelidia, and mentum in *Phalaenopsis*, we also further analyzed MADS-box gene expression profiles by using semi-quantitative RT-PCR (Fig. 9). These results are described in more detail below.

### MADS box A-class genes

Two copies of *FUL*-*like* genes could be detected in the transcriptomes of *P. equestris* and *P. pulcherrima.* These were identified as *ORAP13* and *ORAP11*, which represent *FUL* clade 1 and *FUL* clade 2 lineages (Additional file 3: Fig. S1). No representative of *FUL* clade 3 could be detected, which is consistent with previous findings [64]. Both *ORAP13* and *ORAP11* were highly expressed in early stage floral buds, and their expression decreased in later developmental stages (Fig. 7).

### MADS box B-class genes

One copy of *PI* and four copies of *AP3* gene lineages were detected in the transcriptomes of *P. equestris* and *P. pulcherrima* (Additional file 3: Figs. S2 and S3). There was no expression of *AP3* clade 3 (*PeMADS3* and *PpMADS3*) in the early floral bud, mature callus and gynostemium of *P. equestris* and *P. pulcherrima.* The *AP3* clade 1 gene lineage *PeMADS2* was only expressed in the mature callus of *P. equestris*, while the ortholog *PpMADS2* was expressed in the early floral buds, mature callus and gynostemium of *P. pulcherrima. AP3* clade 2 gene lineages were expressed in early floral buds and mature gynostemium of both *Phalaenopsis* species. *AP3* clade 4 gene lineages *PeMADS4* and *PpMADS4* were highly expressed in all floral structures of both *Phalaenopsis* species and a similar expression pattern was found for the *PI* lineages *PeMADS6* and *PpMADS6* (Fig. 9).

### MADS box C and D-class genes

Three copies of AG and two copies of *STK* could be detected in the transcriptomes of *P. equestris* and *P. pulcherrima*. *PeMADS* 1, *LOC110032491* and *LOC110029636* were found to belong to *AG* clades 1, 2 and 3, whereas *PeMADS7* and *LOC110021571* were found to belong to *STK* clades 1 and 2, respectively (Additional file 3: Figs. S4 and S5). *AG*-like and *STK* gene lineages were mostly expressed in the mature gynostemium of *P. equestris*. In *P. pulcherrima,* the expression of these gene lineages was more varied. Low expression of *AG* and *STK* genes was found in early floral buds and the mature callus of both *P. equestris* and *P. pulcherrima*, except for *AG* clade 3 and *STK* clade 2 gene lineages, that were found to have a higher expression in early stage floral buds of both species (Fig. 7).

### MADS box E-class genes

Four copies of *SEP*-like genes could be detected in the transcriptomes of *P. equestris* and *P. pulcherrima.* In *P. equestris, PeSEP2* was found to be a member of clade 1 and *PeSEP1* a member of clade 2, while *PeSEP3* and *PeSEP4* were found to be members of clade 3 and 4, respectively. *PpSEP1*, *PpSEP3*, *PpSEP2* and *PpSEP4* could be assigned to clades 1–4 (Additional file 3: Fig. S7).

The expression of the *SEP*-like gene lineages varied among both species, the different developmental stages and the different floral structures investigated. The *SEP*-like gene lineage *PeSEP2* was expressed in early floral buds, mature callus and gynostemium of *P. equestris.* However, there was no expression of its ortholog *PpSEP1* in the early floral buds, mature callus and gynostemium of *P. pulcherrima.* The *SEP*-like gene lineages showed similar expression patterns: *SEP* clade 2, 3, and 4 copies were expressed in the early floral buds and mature gynostemium of *P. equestris,* and in the early floral bud, mature callus and gynostemium of *P. pulcherrima* (Figs.  7 and 9).

### MADS box A–E-class genes

*AGL6*-like genes, categorized as AE-class genes [65], were detected with three different copies in both transcriptomes analyzed, in agreement with Cai et al. [66]. The first clade of *AGL6* was found to contain *PeMADS16* and *PpAGL6*-1, the second clade existed of *PeMADS14* and *PpAGL6*-2, and *LOC110023965*/*PeAGL6*-3 were found to be associated with *AGL6* clade 3 (Additional file 3: Fig. S6). The *AGL6*-like copy *PpAGL6*-*1* was expressed in early floral buds, mature callus, and gynostemium. No expression of its ortholog *PeAGL6*-*2* was found in early floral buds, mature callus and gynostemium of *P. equestris. PeAGL6*-3 was highly expressed in early floral buds and mature gynostemium of both species. Its ortholog in *P. pulcherrima* was highly expressed in the callus (Figs. 7 and 9).

## Discussion

### Homology of the callus

**Fig. 10:**
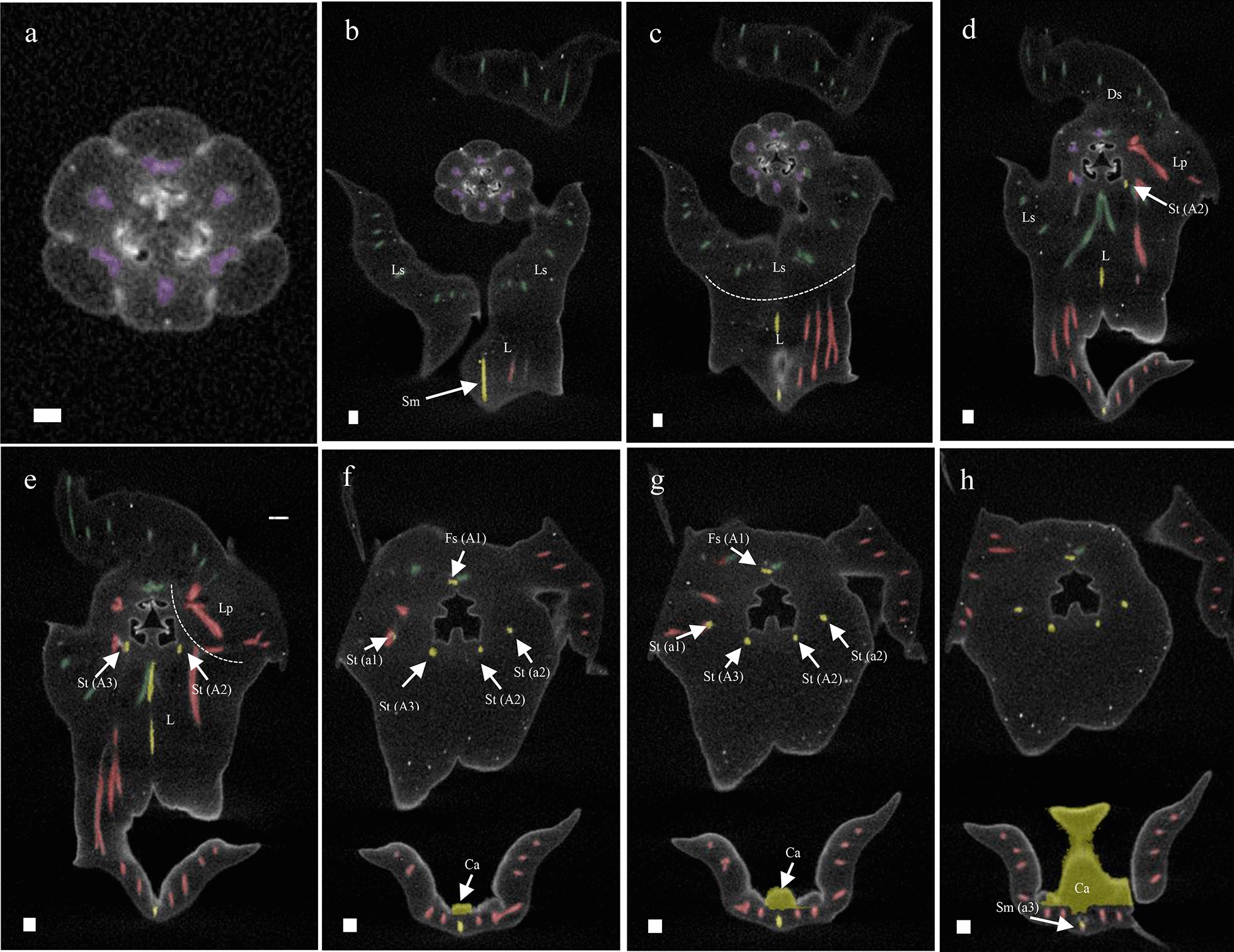
Reconstruction of vascular bundle patterns in a mature flower of *P. equestris* based on μCT scanning. Figures a-h shown in sequence from base to apex. **a** Section through the base of the ovary, showing six vascular bundles indicated in purple; **b** base of receptacle, also showing the vascular bundle in the base of the labellum (L) derived from staminode (Sm) a3 that is feeding the callus and the vascular bundles feeding the lateral sepals (Ls); **c** position where one of the two lateral sepals (Ls) is connecting to the base of the labellum (L); **d** position where the dorsal sepal (Ds), lateral petals (Lp), and labellum (L) connect, showing the first visible staminodal vascular bundle (St/A2); **e** position showing more staminodal (St/A2-A3) vascular bundles, and red vascular bundles feeding the lateral petals (Lp) and labellum (L); **f** position showing the first visible vascular bundles of the fertile stamen (Fs/A1) and four staminodal vascular bundles feeding the stelidia (St/a1-A3 and St/a2-A2); it also shows the first development of the callus (Ca); **g** transversal position of gynostemium showing the vascular bundle running to the fertile stamen (St/A1), and four vascular bundles running to the stelidia (St/a1-A3 and St/a2-A2); **h** position of the labellum with callus (Ca) showing it is supported by a staminodal vascular bundle (Sm/a3). *Ds* dorsal sepal, *Ls* lateral sepal, *Lp* lateral petal, *L* labellum, *Me* mentum, *Ca* callus, *Fs* fertile stamen, *Ss* sterile stamen, *St* stelidia, *A1–A3* staminodes in outer floral whorl, *a1–a3* staminodes in inner floral whorl. Color codes: green = vascular bundles in sepals; red = vascular bundles in petals; yellow = vascular bundles in androecium; purple = vascular bundles in gynoecium. Scale bars: **a** = 0.5 mm; (**b**–**h**) = 100 μm

**Fig. 11:**
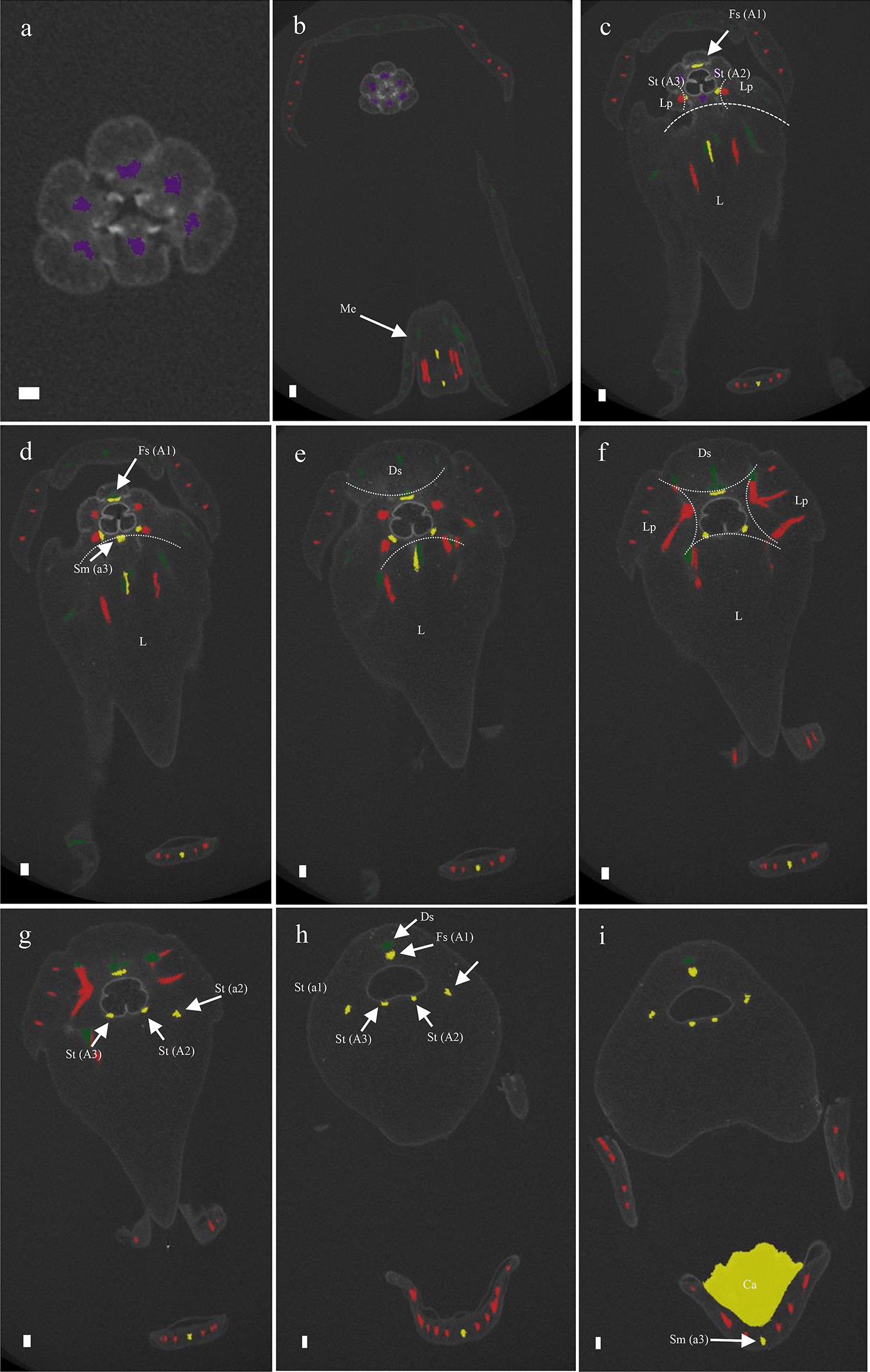
Reconstruction of vascular bundle patterns in a mature flower of *P. pulcherrima* based on μCT scanning. Figures a–i shown in sequence from base to apex. **a**. Section through the base of the ovary, showing six vascular bundles indicated in purple; **b**. base of the receptacle, also showing the vascular bundles in the base of the mentum (Me) derived from the lateral sepals indicated in green, lateral petals indicated in red and staminode a3 indicated in yellow; **c**. position where the labellum connects to the receptacle, showing the vascular bundles that are feeding the fertile stamen (Fs/A1) and stelidia (St/A2-A3) indicated in yellow, and two red vascular bundles feeding the lateral petals (Lp) and labellum (L); **d**. further development of fertile stamen (Fs) and staminodal (Sm/a3) vascular bundles; **e**–**f**. position where dorsal sepal (Ds), lateral petals (Lp) and labellum (L) are merging with the receptacle. **g**. Position where the first vascular bundles that feed the stelidia (St/A2-a2 and St/A3) become visible in the gynostemium; **h**. transversal section through the gynostemium showing the vascular bundles running towards the dorsal sepal (Ds), fertile stamen (Fs), and the stelidia (St/A2-a2 and St/A3-a1); **i**. section through the labellum showing that the callus is supported by the vascular bundle running towards staminode a3 (Sm/a3). *Ds* dorsal sepal, *Ls* lateral sepal, *Lp* lateral petal, *L* labellum, *Me* mentum, *Ca* callus, *Fs* fertile stamen, *Ss* sterile stamen, *St* stelidia, *A1*–*A3* staminodes in outer floral whorl, *a1–a3* staminodes in inner floral whorl. Color codes: green = vascular bundles in sepals; red = vascular bundles in labellum and petals; yellow = vascular bundles in androecium; purple = vascular bundles in gynoecium. Scale bars: **a** = 0.5 mm; **b**–**i** = 100 μm

Mature flowers of *P. equestris* and *P. pulcherrima* were used to produce micro 3-D CT scans to study their anatomy in more detail. The vascular bundles were stained with PTA to be able to study their orientation. The callus was found to be fed by one staminodial (a3) and several petaloid vascular bundles. The staminodial vascular bundle, connected to the base of the callus, apparently fed the callus to develop and grow in size since no bundles were detected inside the callus. Its first appearance occurs during the second stage of floral development. A small callus is located on the base of the labellum, eventually guarded by the lateral lobes of the labellum that are still developing during this stage. The callus grew significantly during all developmental stages, reaching an approximate size of 4 mm in diameter in mature flowers. The development of the callus of *E. pusilla* and *P. equestris* was highly similar. A different pattern was found for the development of the callus of *P. pulcherrima*: in early development, the size ratio between the labellum and callus was 2:1, whereas in later stages, the ratio between labellum and callus became 3:1, indicating more expansion of the labellum as compared to the callus (Figs. 10, 11).

Because of the mixed petaloid-staminodial origin of the callus found in our micro-CT analyses, we expected a combined expression of MADS-box A, B, and E-class genes in this particular floral organ. Expression of MADS box AE-class *AGL6*-clade 2, and B-class *PI*-like and *AP3*-like clade 4 copies was indeed found in the mature callus of both species of *Phalaenopsis* investigated (Fig. 9a). The observed expression of *PI*-like genes is in agreement with other studies that report this gene to be involved in the regulation of sepals, petals, labellum and gynostemium in *Phalaenopsis* and *Dendrobium* [27, 45, 67]. Differences in expression of certain gene copies among the two species of *Phalaenopsis* investigated were found as well. The *SEPALLATA* E-class MADS-box gene *PeSEP2* was expressed in the large callus of *P. equestris*, but its ortholog *PpSEP1* was not expressed in the small callus of *P. pulcherrima*. Expression of the AE-class *AGL6* clade 1 and *AGL6* clade 3, B-class *AP3* clade 1 and *AP3* clade 2, and E-class *SEP* clade 2, 3 and 4 copies was only observed in the small callus of *P. pulcherrima* but not in the large callus of *P. equestris*. These results agree with a previous study that found that the *Phalaenopsis* B-class *AP3* and *PI*-like genes and AE-class *AGL6* genes may form higher protein complexes with several *PeSEP* gene copies to determine floral organ identity [28]. We found higher expression of *DIV* 2 and lower expression of *DIV* 1 copies in the larger callus of *P. equestris* than in the smaller callus of *P. pulcherrima*.

### Homology of the stelidia and mentum

The stelidia develop differently in comparison to the callus in both *P. equestris* and *P. pulcherrima*. Four vascular bundles can be found inside the stelidia that are derived from the merged staminodes a1 plus A3 and a2 plus A2 [68]. In later developmental stages, the gynostemium develops by fusion of the fertile stamen A1, median carpel, and staminodes, which were still separate floral structures in early developmental stages. In some species, such as *P. equestris*, staminodial tissue fuses with the gynostemium in an earlier stage than in other species. In *P. pulcherrima*, the staminodial tissue remains separate and increases in size during later developmental stages. Our findings support those of Kurzweil and Kocyan [24], who found that staminodes a1 and a2 are almost always present during early ontogeny of monandrous orchids, but become incorporated in various degrees in the gynostemium in later stages. The staminodes either remain visible as stelidia or become incorporated in the gynostemium (Fig. 11).

Our ontogenetic study showed that a mentum develops quite late in *P. pulcherrima* as it started to develop only in phase 4 (Fig. 11). This result is congruent with observations obtained for other orchids with a mentum. For example, in *Bletia purpurea*, where the mentum was described as an organ originating from a basal extension of the gynostemium and fusion with staminodes and lateral sepals [24, 69]. Our micro-CT results provide further support for this hypothesis as both staminodial (a3, A2 and A3) and sepaloid vascular bundles were found to be feeding the mentum, next to vascular bundles also feeding the labellum and petals.

Because of the staminodial origin of the stelidia, we expected expression of MADS-box C-, D- and E-class genes in these floral structures as previously found in the stelidia of *Erycina pusilla* [31]. Expression of MADS box AE-class *AGL6* clade 2 and 3 genes was indeed detected in these structures together with E-class *SEP* clade 2,3 and 4 lineages (Fig. 9b). Because of the mixed petaloid, sepaloid, staminodial origin of the mentum, we expected expression of all five classes of MADS-box genes in this organ. As expected, B-class *AP3* clade 3 and 4 and *PI*-like genes were expressed in the gynostemium of both species of *Phalaenopsis* investigated. Expression of MADS box B-class *AP3* clade 1 and AE-class *AGL6* clade 1 copies was detected in the gynostemium with pronounced stelidia and mentum of *P. pulcherrima*, whereas these gene copies were not expressed in the gynostemium of *P. equestris*, lacking a pronounced mentum and stelidia. In the gynostemium of the latter species, expression of the E-class *SEP* clade 1 gene lineage was higher than in the gynostemium of *P. pulcherrima*. In both species of *Phalaenopsis* investigated, *SEP* clade 1 copies were found to be co-expressed with up to two different copies of *AGL*6-like genes, whereas the other three *SEP* lineages were co-expressed with all three *AGL*6-copies. This finding is in line with multiple studies that hypothesize that *SEP* clade 1 and *AGL6*-like genes once had a common function that was conserved during evolution [47, 70–72]. Of the MYB and TCP transcription factors analyzed, no differential expression could be detected in the stelidia and mentum of the two species of *Phalaenopsis* analyzed.

### Implications for current orchid floral models

We found that most of the MADS-box AE-class *AGL6*-like, B-class *AP3*-like and AE-class *SEP*-like gene lineages investigated were expressed in early floral buds of both *Phalaenopsis* species investigated. These results are in line with previous findings of *AP3* gene copies being involved in orchid floral primordia development [36]. Similarly, *AGL6*-like *OSMADS6* was reported to have a function in regulating early meristem identity and floral development in rice [73]. During later development, these genes showed differential expression in separate floral organs.

**Fig. 12:**
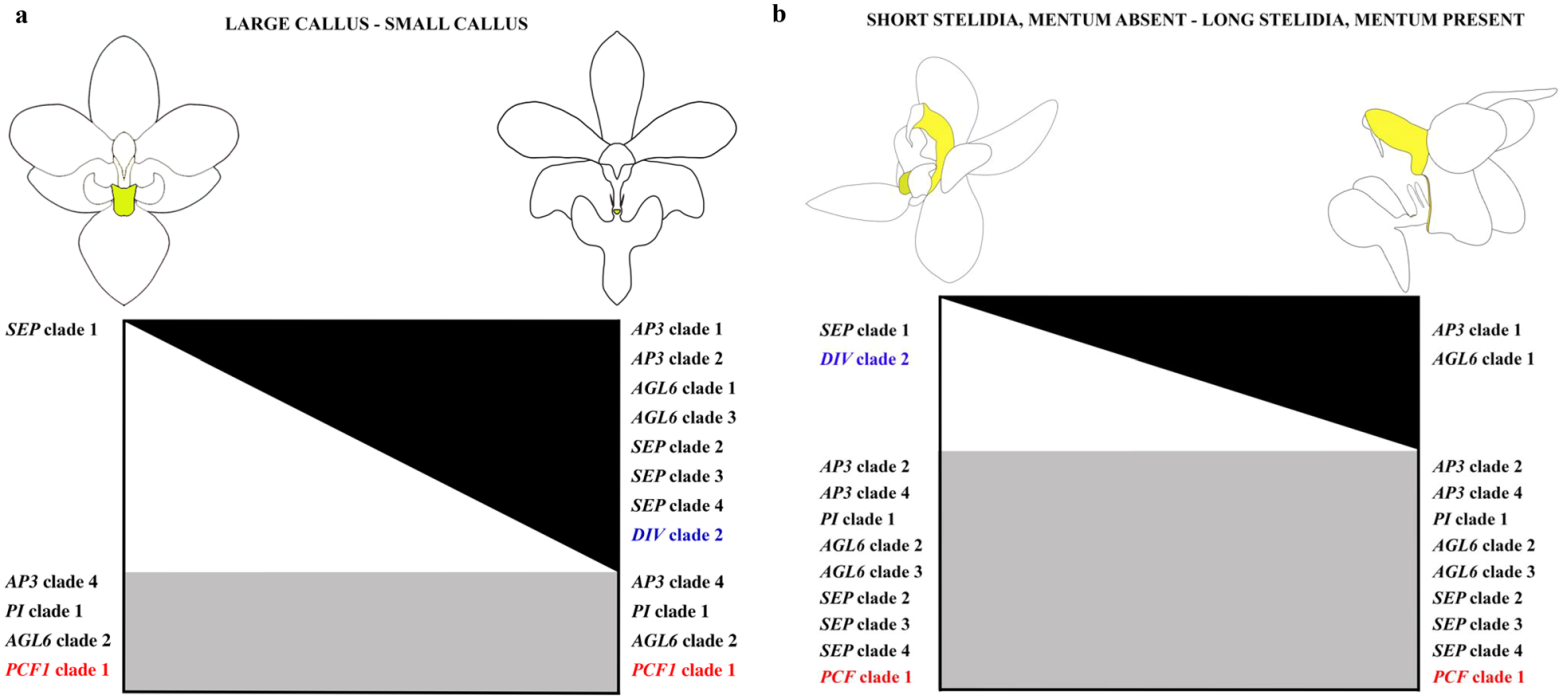
Summary of expression of MADS-box (black), MYB (blue), and TCP (red) genes involved in the differentiation of callus, stelidia and mentum (all indicated in yellow) of *P. equestris* and *P. pulcherrima*. **a** Expression of *SEP* clade 1 genes is high (*indicated on the left side of the* *white triangle*) in the large callus of *P. equestris* (*left side*) but absent (*indicated on the right side of the* *white triangle*) in the small callus of *P*. *pulcherrima* (*right side*). Expression of *AP3* clade 1, *AP3* clade 2, *AGL6* clade 1, *AGL6* clade 3, *SEP* clade 2, *SEP* clade 3, *SEP* clade 4, and *DIV* clade 2 genes is high (*indicated on the right side of the* *black triangle*) in the small callus of *P. pulcherrima* (*right side*) but absent (*indicated on the left side of the* *black triangle*) in the large callus of *P. equestris* (*left side*). Expression of *AP3* clade 4, *PI* clade 1, *AGL6* clade 2, and *PCF* clade 1 genes is high (*indicated by the grey box*) in the callus of both *Phalaenopsis* species. **b** Expression of *SEP* clade 1 and *DIV* clade 2 genes is high (*indicated on the left side of the white triangle*) in the short stelidia and absent mentum of *P. equestris* (*left side*) but absent (*indicated on the right side of the* *white triangle*) in the long stelidia and pronounced mentum of *P. pulcherrima* (*right side*). Expression of *AP3* clade 1, and *AGL6* clade 1 genes is high (*indicated on the right side of the* *black triangle*) in the long stelidia and pronounced mentum of *P. pulcherrima* (*right side*) but absent (*indicated on the left side of the* *black triangle*) in the short stelidia and absent mentum of *P. equestris* (left side). Expression of *AP3* clade 2, *AP3* clade 4, *PI* clade 1, *AGL6* clade 2, *AGL6* clade 3, *SEP* clade 2, *SEP* clade 3, *SEP* clade 4, and *PCF* clade 1 genes is high (*indicated by the grey box*) in the stelidia and mentum of both *Phalaenopsis* species. Illustrations by Richa Kusuma Wati

In our study, we discovered possible new roles of B-class and *AGL6*-like gene lineages in the formation of the callus, mentum and stelidia in orchid flowers. We found that the SP complex *PpAP3*-*1*/*PpAGL6*-*1/PpPI,* that promotes sepal and petal development in the perianth, is also expressed in the callus, mentum and stelidia (Fig. 12). *AGL6* proteins can interact with several MADS proteins during floral development [29, 47, 72, 74]. Our results suggest that duplication of *AGL6* might have led to novel functions of these genes in the flowers of *Phalaenopsis*, especially regarding the development of the stelidia and mentum. Combined expression of the SP complex genes together with *SEP* clade 2, 3 and 4 lineages, but lack of expression of the *SEP* clade 1 lineage, results in a small callus, pronounced mentum and long stelidia in *P. pulcherrima*. The reverse, namely the expression of the *SEP* clade 1 gene lineage but lack of expression of the SP complex and *SEP* clade 2, 3 and 4 gene lineages, results in a large callus in *P. equestris*. The expression of all four *SEP* lineages but lack of expression of the SP complex results in the lack of a mentum and short stelidia in *P. equestris*. No expression of *AP3* clade 3 lineages was detected in either the callus or gynostemium of both species of *Phalaenopsis* investigated. We therefore hypothesize that the role of the *AP3* clade 3 lineage, one of the three gene lineages active in the L-complex, may be replaced by the *AP3* clade 4 lineage in the callus and by *AP3* clade 2 and/or 4 in the gynostemium.

We detected similar expression patterns of E-class *SEP* clade 2, 3, and 4 lineages in the callus and gynostemium of both species of *Phalaenopsis* investigated (Fig. 10). In *Arabidopsis thaliana* these lineages have highly redundant functions [75, 76] but the role of the individual *SEP* genes in the floral organs and structures of *Phalaenopsis* seems very different. Pan et al. [28] discovered that downregulation of the *SEP* clade 1 lineage in *P. equestris* did not produce any morphological effect, in contrast with downregulation of the *SEP* clade 3 lineage, which resulted in a change in floral morphology. Pelaz et al. [75] reported that downregulation of the activity of *SEP1*, 2, and 3 results in all organs developing into sepals in *Arabidopsis thaliana.* This implies that *SEP* genes are required for the development of petals in the second, stamens in the third and carpels in the fourth floral whorl. Our results suggest that these genes are also involved in the regulation of the first, second and third whorl mentum, second whorl callus and third whorl stelidia in the flowers of *Phalaenopsis*.

Zygomorphy of flowers improves the efficiency of their pollination. Bilateral symmetry ensures that pollinators can only make contact with the stamen and stigma from one particular direction for pollen removal and deposition. This makes the placement and removal of pollen more precise [77]. In orchid flowers, zygomorphy involves multiple structures, both in the outer two floral whorls and the inner three floral whorls. Part of these structures are of mixed origin such as the mentum, made up of fused sepal, petal and staminodial parts, and the remainder of the gynostemium, made up of fused staminodes, stamens and the stigma. MYB and TCP transcription factors code for floral zygomorphy. The involvement of *MYB* and *TCP* genes in orchid floral symmetry has previously been examined in *Orchis italica*, *Dendrobium catenatum*, *Cattleya triana* and *Phalaenopsis equestris* [40, 41, 78, 79] but none of these studies investigated differential expression among the callus, mentum and stelidia. Expression of *PCF* clade 1 was detected in the early floral buds, and mature callus and gynostemium of both *Phalaenopsis* species. This finding is similar to that of Li et al. [80], who detected expression of *PePCF8* gene, a *PCF* clade 1 lineage, in floral organs and vegetative tissue of *P. equestris*. To our knowledge, we are the first to report the expression of *DIV* 2 in the small labellar callus of *P. pulcherrima* and short stelidia and gynostemium lacking a mentum of *P. equestris.* Interestingly, there appears to be a trade-off between *DIV* clade 2 and *SEP* clade 1 expression in the callus of *Phalaenopsis*. Similarly, a trade-off seems present between the expression of *DIV* clade 2 and *AP3* clade 1 and *AGL6* clade 1 in the stelidia and mentum. Follow-up studies using qPCR and functional analyses can shed more light on the genetic regulation of the callus, stelidia, and mentum.

## Conclusions

Evolution and development of [1] the callus on the labellum, [2] the stelidia on the lateral sides of the gynostemium, and [3] the mentum at the base of the gynostemium, labellum and adaxial sepals were studied in two species of *Phalaenopsis* with very differently shaped flowers. In both *P. equestris* and *P. pulcherrima*, the callus first emerges in developmental stage 1, and it stops growing in stage 3, with a more pronounced growth in *P. equestris* as compared with *P. pulcherrima*. In both species, vascular bundles were found feeding the callus at the position of the staminode in the outer whorl. Considering the MADS-box genes, expression of *SEP* clade 1 gene copies was related to the larger sized callus of *P. equestris.* Expression of *AP3*-*4/PI*/*AGL6*-*2* gene copies found in the callus of both species further supports a staminodial origin of this particular tissue, as also found earlier for the emergent orchid model system *Erycina pusilla*. Of the MYB and TCP transcription factors analyzed, *DIV* clade 2 lineages were found to be highly expressed in the small callus of *P. pulcherrima* but not in the large callus of *P. equestris. PCF* clade 1 lineages were found to be highly expressed in the callus of both *Phalaenopsis* species investigated.

In both species, the stelidia emerge as separate structures in stage 2. In *P. equestris*, they stop elongating in stage 3 whereas in *P. pulcherrim*a the stelidia continue to elongate up to stage 5. In both species, vascular bundles were found feeding the stelidia at the position of the outer and inner whorl stamens. Expression of *AP3* clade 2 and 4, *PI, AGL6* clade 2 and 3, and *SEP* clade 2,3 and 4 gene lineages found in the stelidia of both species further supports a staminodial origin of these particular structures, as also found earlier for the emergent orchid model system *E. pusilla*. *DIV* clade 2 lineages were found to be highly expressed in short stelidia *P. equestris* but not in the long stelidia of *P. pulcherrima.*

In *P. pulcherrima*, the mentum first emerges as separate organ in developmental stage 4 so quite late during floral ontogeny. Part of the vascular bundles feeding the sepals, petals and staminodes in *P. equestris* were found to run towards this organ. A possible sepaloid-petaloid-staminodial origin was further confirmed by the expression of SP complex *PpAP3*-*1*/*PpAGL6*-*1/PI* genes in this floral organ. To our knowledge, our study is the first to find combined micro-morphological and molecular evidence for a possible sepaloid-petaloid-staminodial origin of the orchid mentum. Transformation studies should be carried out to find proof for this hypothesis. *DIV* clade 2 lineages were found to be highly expressed in the gynostemium without a mentum of *P. equestris* but not in the pronounced mentum of *P. pulcherrima. PCF* clade 1 lineages were found to be highly expressed in the gynostemium of both *Phalaenopsis* species investigated.

In summary, we detected differential growth and expression of MADS box *AP3/PI*-like, *AGL*6-like and *SEP*-like, and MYB *DIV*-like gene copies in the callus, stelidia and mentum of two species of *Phalaenopsis,* of which these floral structures are very differently shaped and sized. By unraveling the first developmental genetic factors driving the floral diversity of this genus, we have come a step closer to understanding how different fits between flowers and small and larger pollinators evolved that ultimately gave rise to reproductive isolation and eventually diversification in these orchids.

## Supplementary information

**Additional file 1: Table S1.** List of primers used for semi quantitative RT PCR analysis. **Table S2.** List of Phalaenopsis sequences generated from transcriptome analysis. **Table S3.** List of sequences used for phylogenetic analyses. **Table S4.** Results of differential gene expression of MADS-box gene analyses using DESeq in R. **Table S5.** Results of differential gene expression of MYB and TCP genes analyses using DESeq in R.

**Additional file 2: Movie S1.** 3D visualization of vascular bundle patterns in a mature flower of *P. equestris* based on μCT scanning. **Movie S2.** 3D visualization of vascular bundle patterns in a mature flower of *P. pulcherrima* based on μCT scanning.

**Additional file 3: Figure S1.** Maximum likelihood tree of the FUL subfamily. **Figure S2.** Maximum likelihood tree of the AP3 subfamily. **Figure S3.** Maximum likelihood tree of the PI subfamily. **Figure S4.** Maximum likelihood tree of the AG subfamily. **Figure S5.** Maximum likelihood tree of the STK subfamily. **Figure S6.** Maximum likelihood tree of the AGL6 subfamily. **Figure S7.** Maximum likelihood tree of the SEP subfamily. **Figure S8.** Maximum likelihood tree of the DIV subfamily. **Figure S9.** Maximum likelihood tree of the RAD subfamily. **Figure S10.** Maximum likelihood tree of the DRIF subfamily. **Figure S11.** Maximum likelihood tree of the TCP family.

## Data Availability

All supporting data are available in Additional files.
