# Supplementary material for: Evolution and development of three highly specialized floral structures of bee-pollinated Phalaenopsis species
Source: EvoDevo. 2020 Aug 10;11:16. doi: 10.1186/s13227-020-00160-z (PMC7418404; doi:10.1186/s13227-020-00160-z)
Supplement: Supplementary file 2 — Additional file 2: Movie S1. 3D visualization of vascular bundle patterns in a mature flower of P. equestris based on μCT scanning. Movie S2. 3D visualization of vascular bundle patterns in a mature flower of P. pulcherrima based on μCT scanning. [file 13227_2020_160_MOESM2_ESM.pptx]

## Slide 1
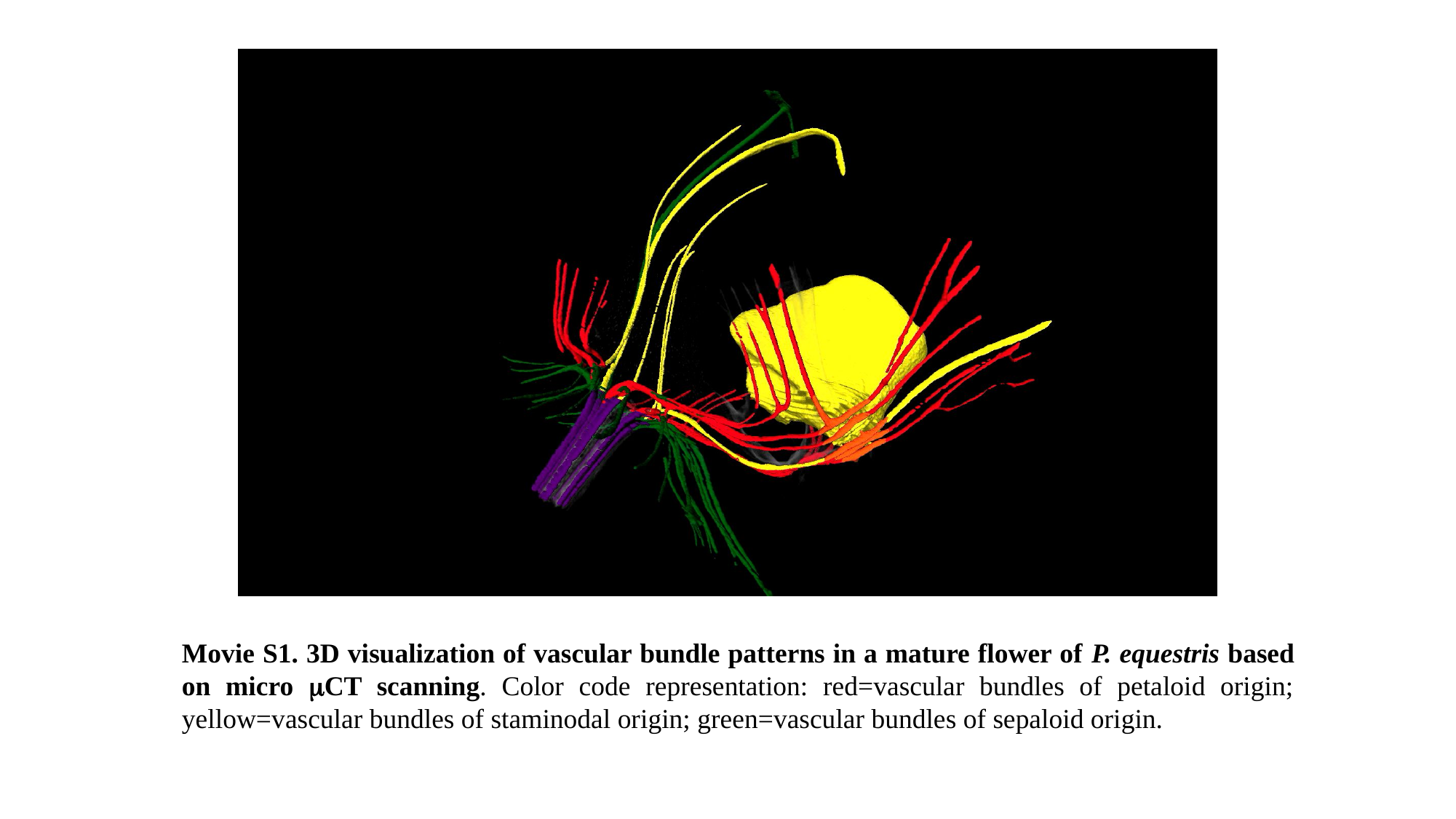

Movie S1. 3D visualization of vascular bundle patterns in a mature flower of P. equestris based on micro CT scanning. Color code representation: red=vascular bundles of petaloid origin; yellow=vascular bundles of staminodal origin; green=vascular bundles of sepaloid origin.

## Slide 2
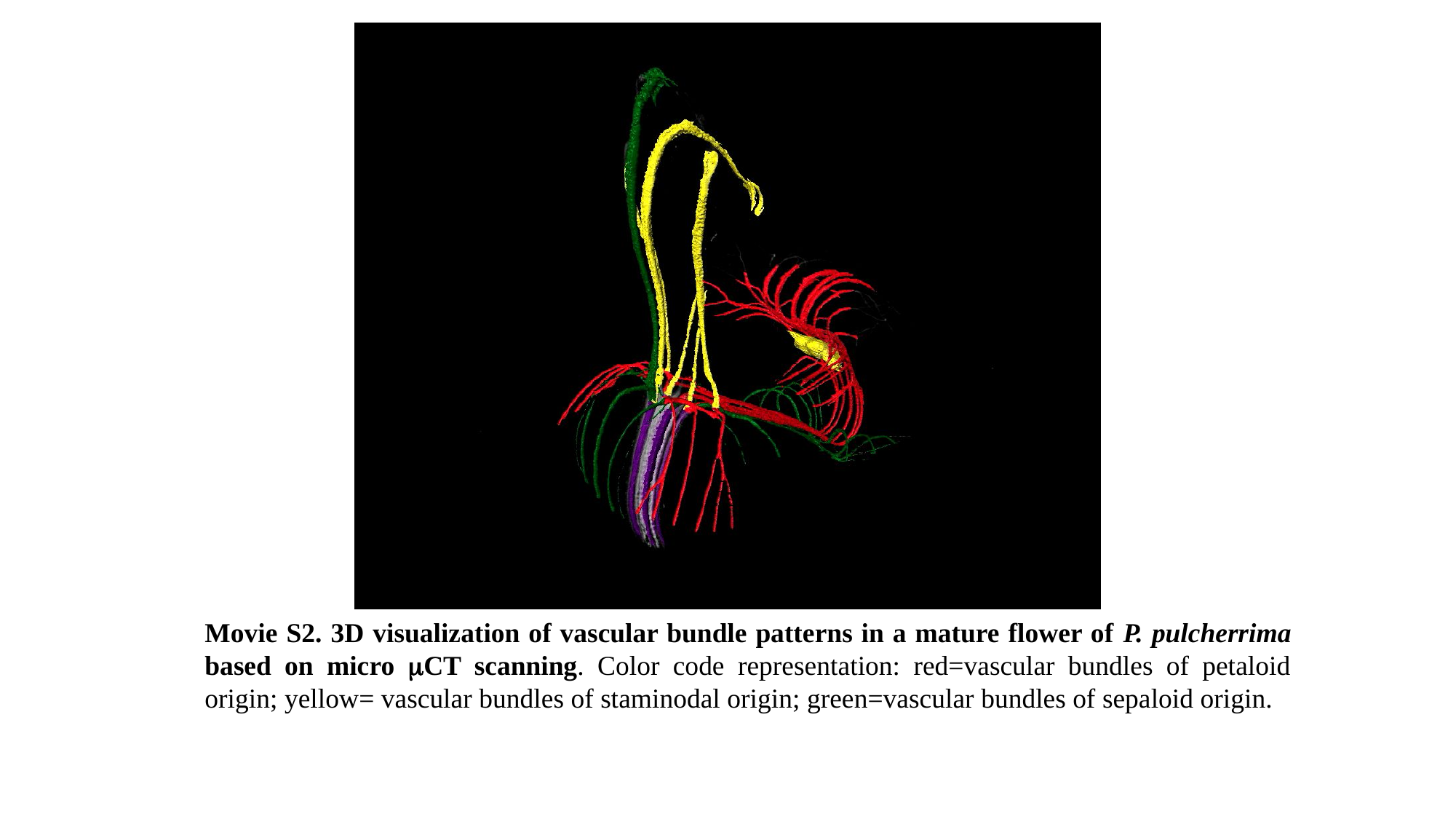

Movie S2. 3D visualization of vascular bundle patterns in a mature flower of P. pulcherrima based on micro CT scanning. Color code representation: red=vascular bundles of petaloid origin; yellow= vascular bundles of staminodal origin; green=vascular bundles of sepaloid origin.
