# Supplementary material for: Evolution and development of three highly specialized floral structures of bee-pollinated Phalaenopsis species
Source: EvoDevo. 2020 Aug 10;11:16. doi: 10.1186/s13227-020-00160-z (PMC7418404; doi:10.1186/s13227-020-00160-z)
Supplement: Supplementary file 3 — Additional file 3: Figure S1. Maximum likelihood tree of the FUL subfamily. Figure S2. Maximum likelihood tree of the AP3 subfamily. Figure S3. Maximum likelihood tree of the PI subfamily. Figure S4. Maximum likelihood tree of the AG subfamily. Figure S5. Maximum likelihood tree of the STK subfamily. Figure S6. Maximum likelihood tree of the AGL6 subfamily. Figure S7. Maximum likelihood tree of the SEP subfamily. Figure S8. Maximum likelihood tree of the DIV subfamily. Figure S9. Maximum likelihood tree of the RAD subfamily. Figure S10. Maximum likelihood tree of the DRIF subfamily. Figure S11. Maximum likelihood tree of the TCP family. [file 13227_2020_160_MOESM3_ESM.docx]

**Supplementary Figures**


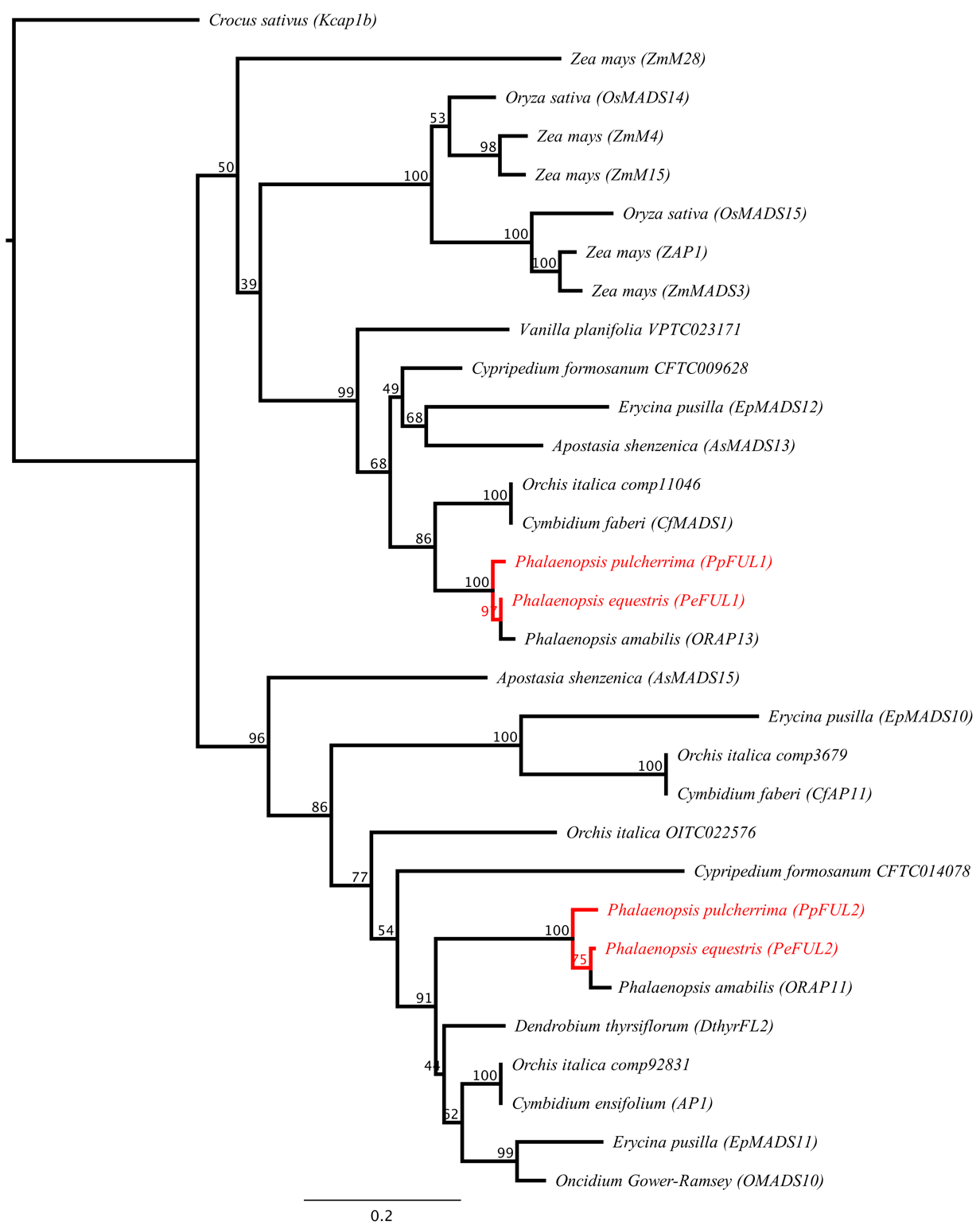


***FUL* clade 1**

***FUL* clade 3**

***FUL* clade 2**

**Figure S1. Maximum likelihood tree of the FUL subfamily.** The phylogenetic tree was constructed with 100 bootstrap replicates based on the alignment of full-length amino acid sequences of the genes and edited by colouring the nodes belonging to all *Phalaenopsis* genes generated in this study red. Numbers above the nodes represent bootstrap values.

***AP3* clade 4**

***AP3* clade 2**

***AP3* clade 3**

***AP3* clade 1**


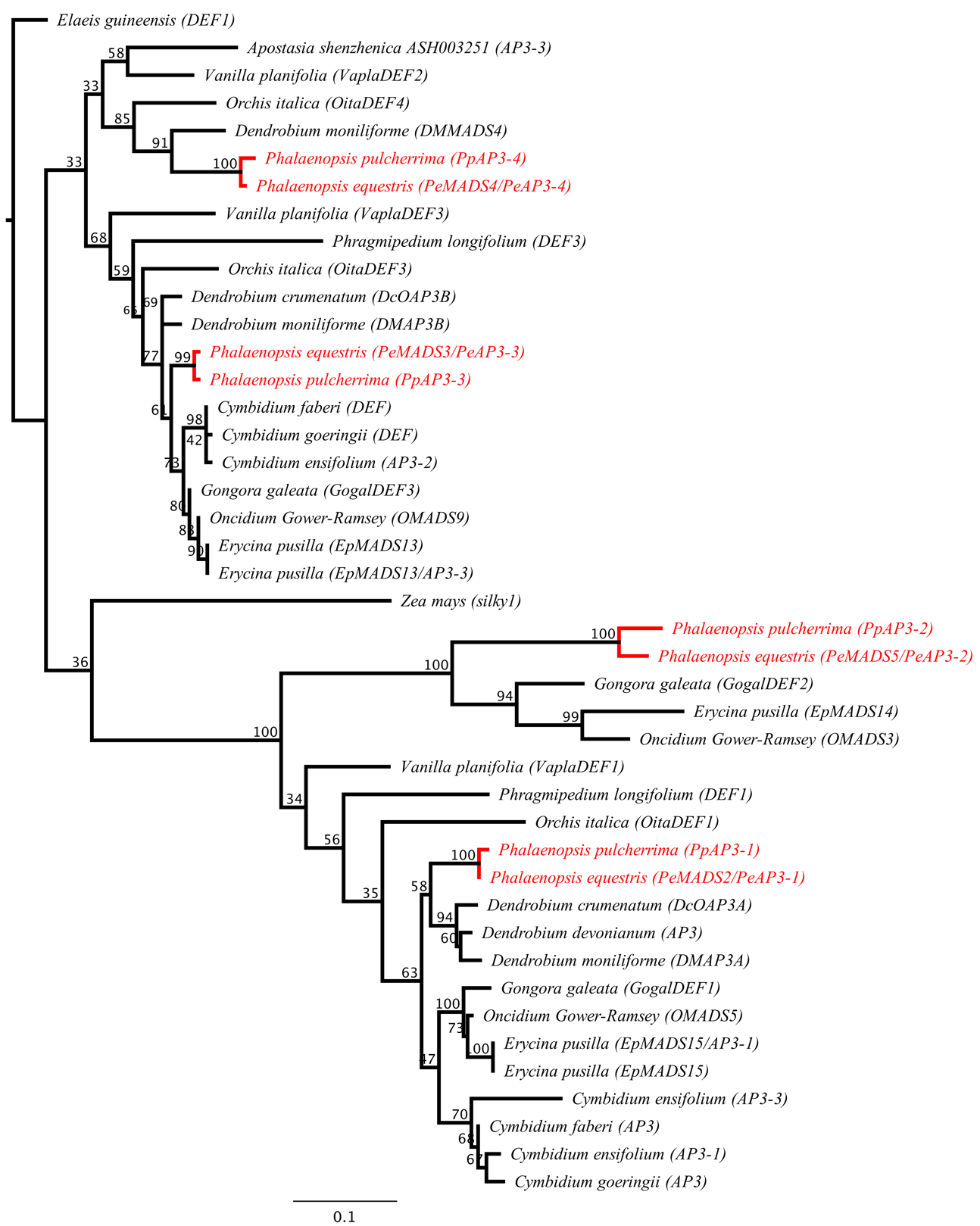


**Figure S2. Maximum likelihood tree of the AP3 subfamily.** The phylogenetic tree was constructed with 100 bootstrap replicates based on the alignment of full-length amino acid sequences of the genes and edited by colouring the nodes belonging to all *Phalaenopsis* genes generated in this study red. Numbers above the nodes represent bootstrap values.

***PI* clade 1**

***PI* clade 2**


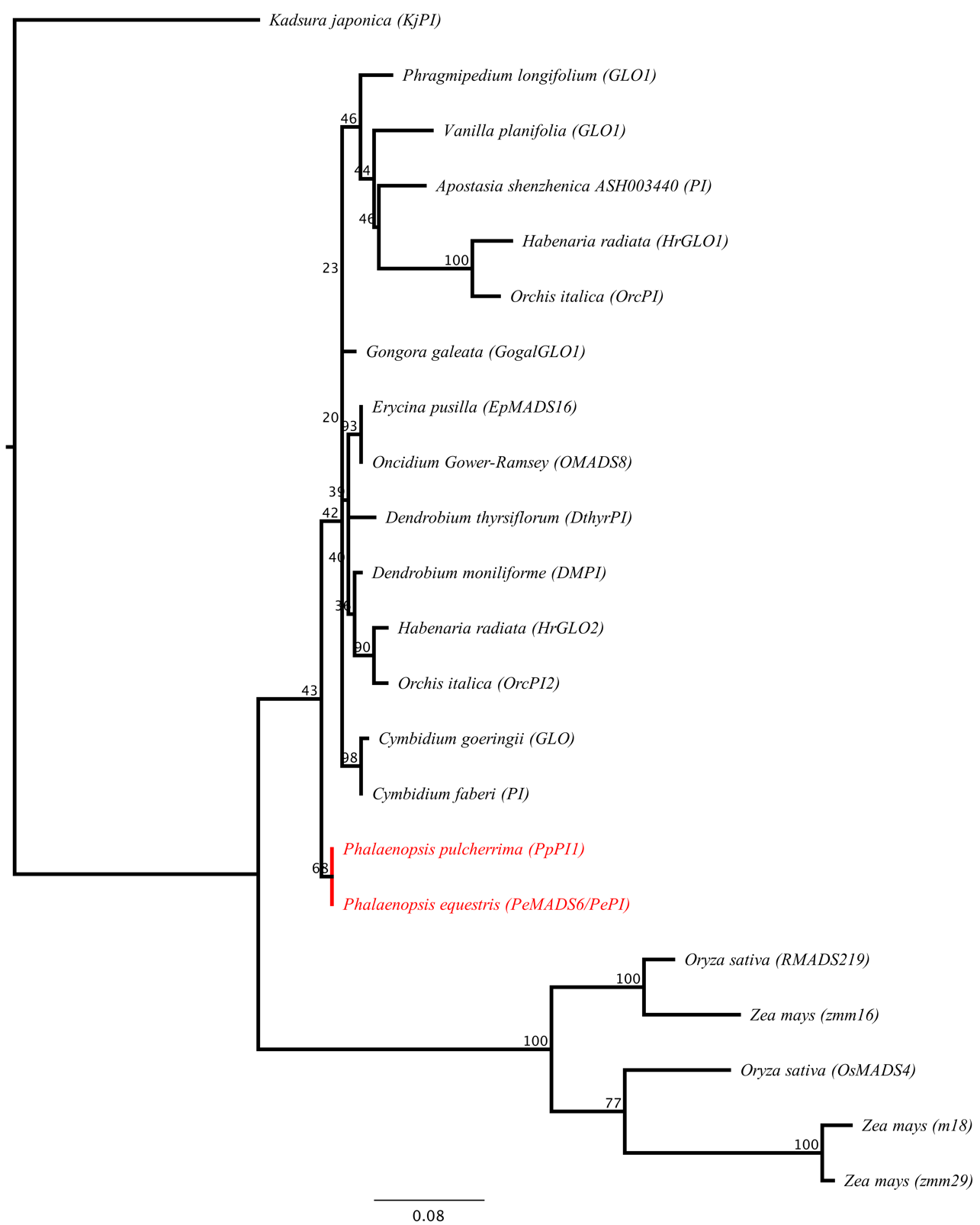


**Figure S3. Maximum likelihood tree of the PI subfamily.** The phylogenetic tree was constructed with 100 bootstrap replicates based on the alignment of full-length amino acid sequences of the genes and edited by colouring the nodes belonging to all *Phalaenopsis* genes generated in this study red. Numbers above the nodes represent bootstrap values.

***AG* clade 2**

***AG* clade 1**

***AG* clade 3**


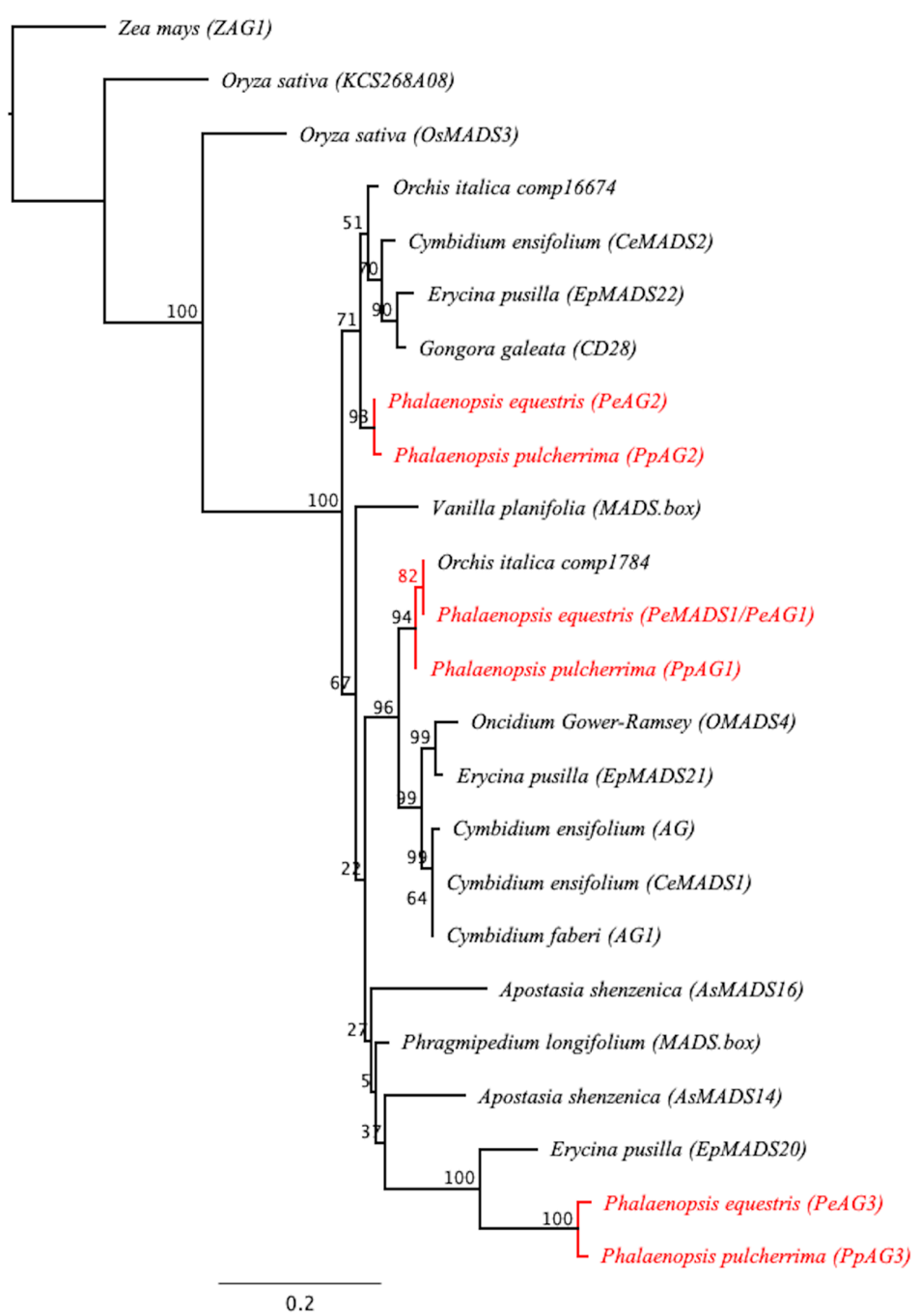


**Figure S4. Maximum likelihood tree of the AG subfamily.** The phylogenetic tree was constructed with 100 bootstrap replicates based on the alignment of full-length amino acid sequences of the genes and edited by colouring the nodes belonging to all *Phalaenopsis* genes generated in this study red. Numbers above the nodes represent bootstrap values.

***STK* clade 2**

***STK* clade 1**


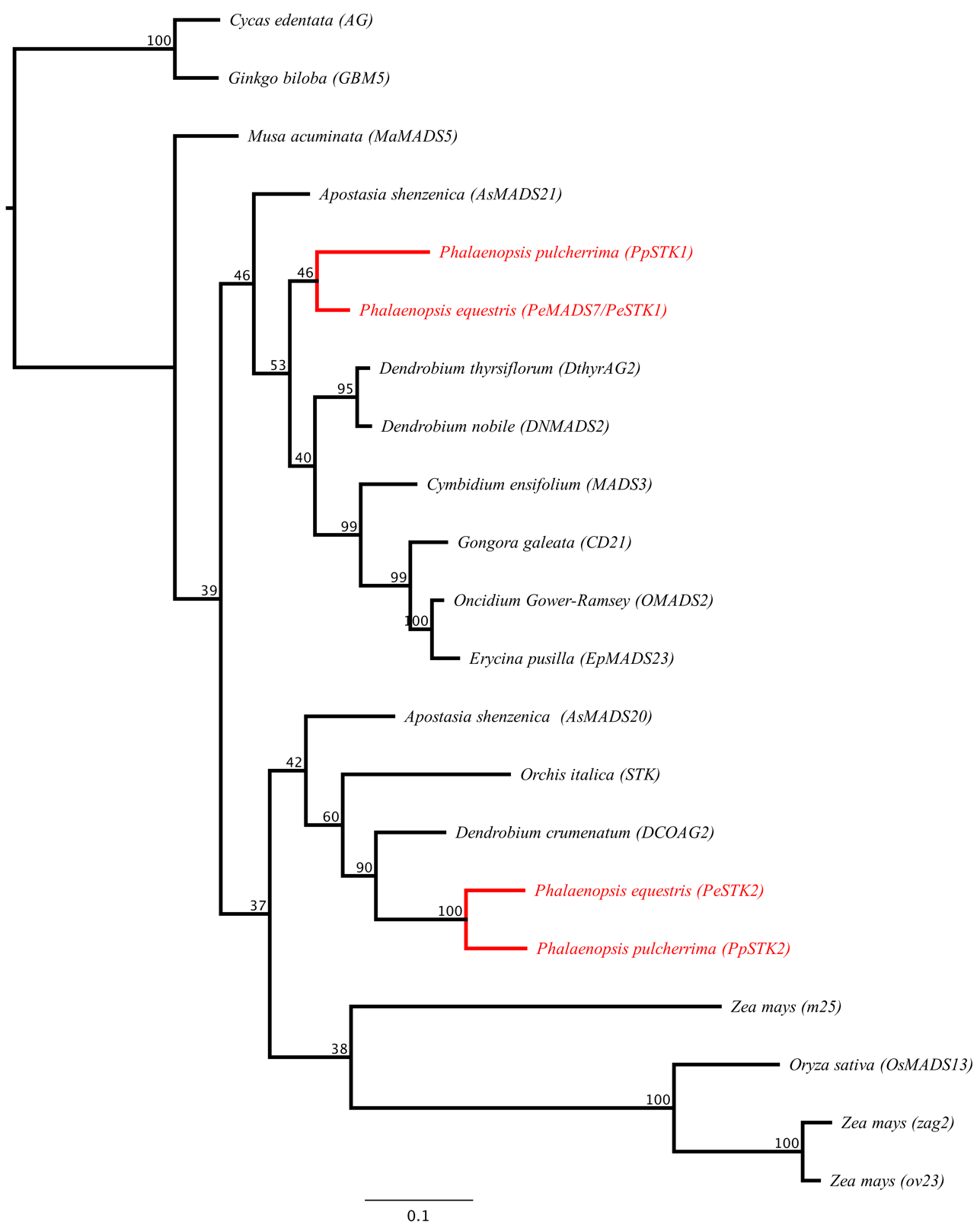


**Figure S5. Maximum likelihood tree of the STK subfamily.** The phylogenetic tree was constructed with 100 bootstrap replicates based on the alignment of full-length amino acid sequences of the genes and edited by colouring the nodes belonging to all *Phalaenopsis* genes generated in this study red. Numbers above the nodes represent bootstrap values.

***AGL6* clade 2**

***AGL6* clade 3**

***AGL6* clade 1**


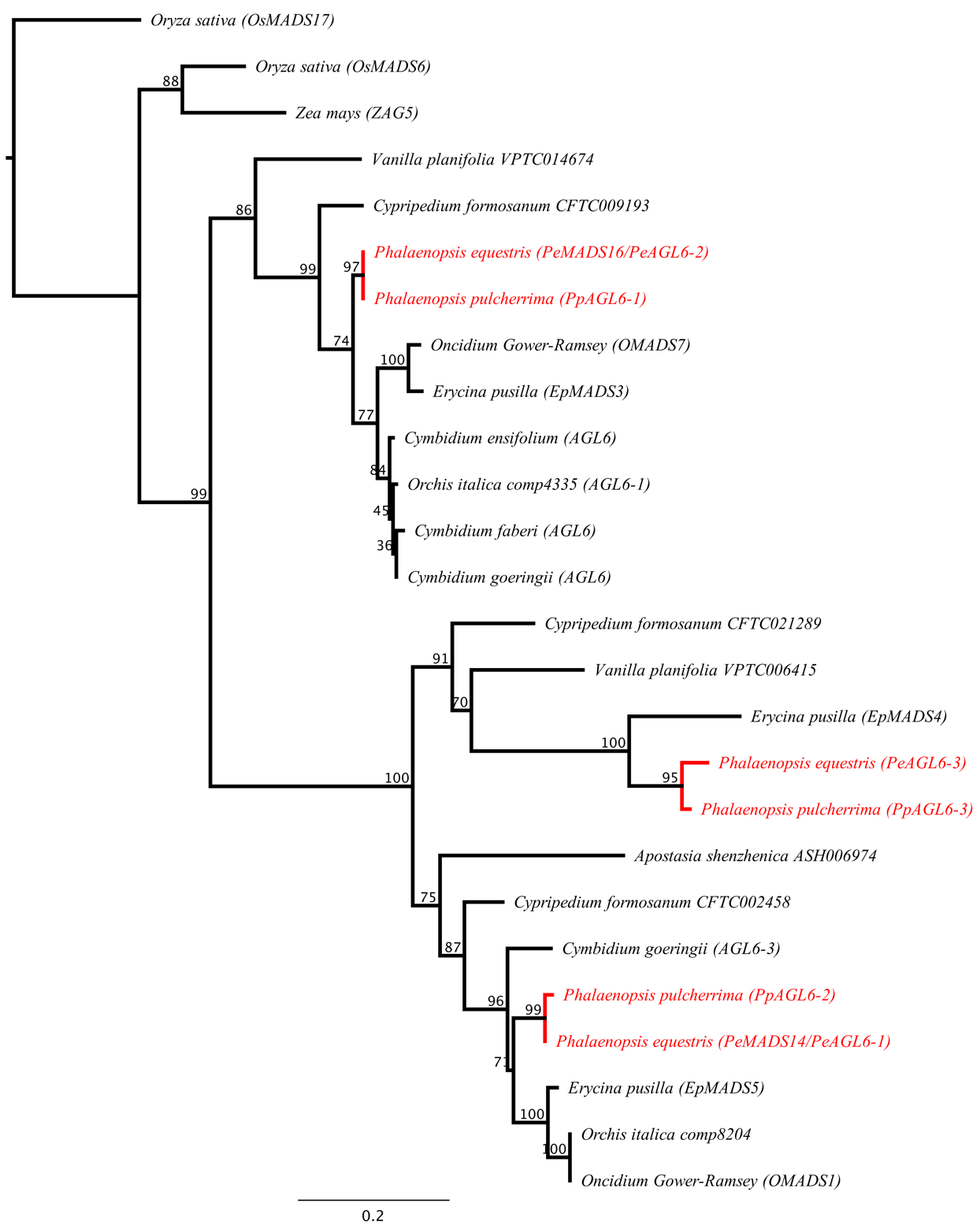


**Figure S6. Maximum likelihood tree of the AGL6 subfamily.** The phylogenetic tree was constructed with 100 bootstrap replicates based on the alignment of full-length amino acid sequences of the genes and edited by colouring the nodes belonging to all *Phalaenopsis* genes generated in this study red. Numbers above the nodes represent bootstrap values.

***SEP* clade 3**

***SEP* clade 2**

***SEP* clade 1**

***SEP* clade 4**


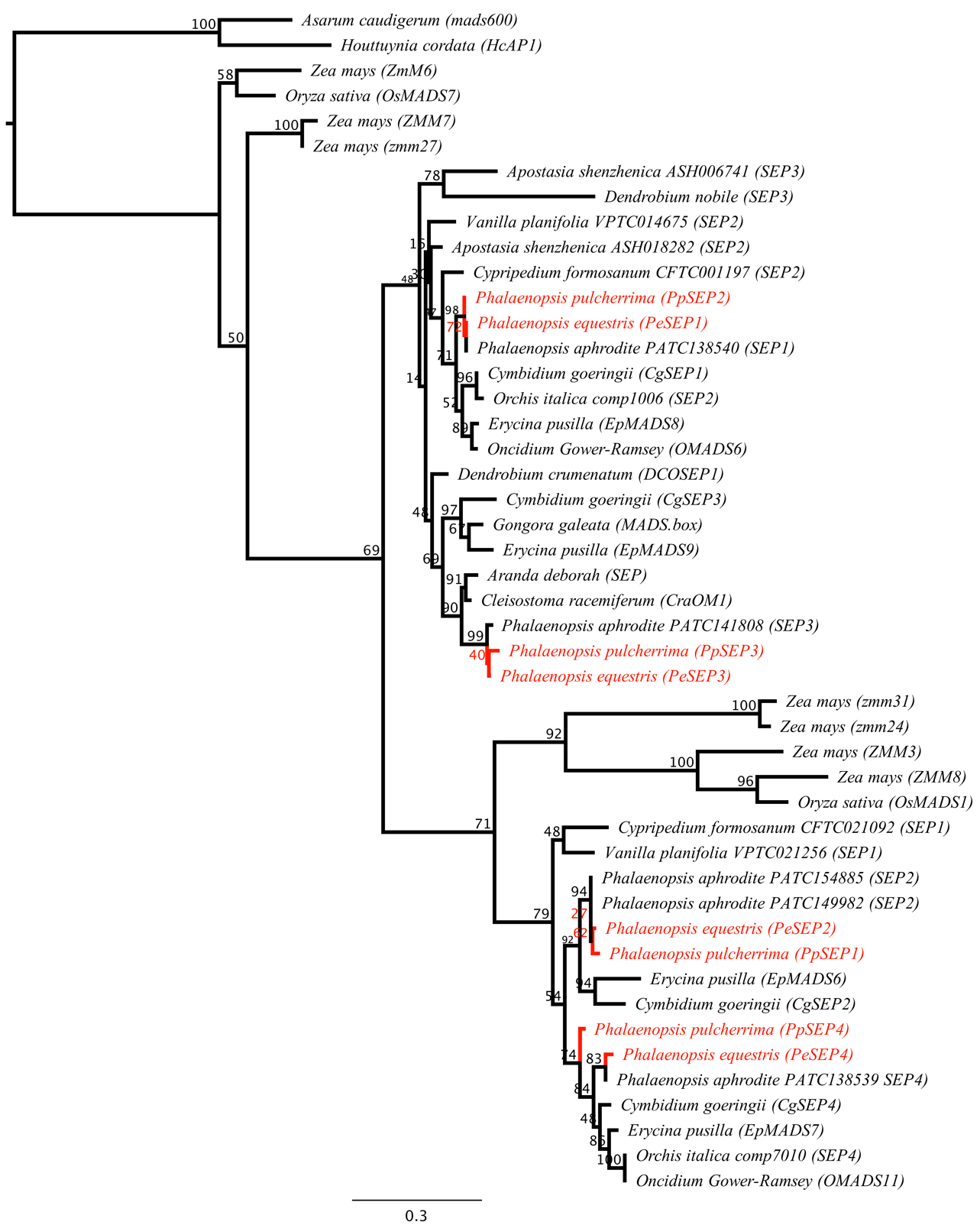


**Figure S7. Maximum likelihood tree of the SEP subfamily.** The phylogenetic tree was constructed with 100 bootstrap replicates based on the alignment of full-length amino acid sequences of the genes and edited by colouring the nodes belonging to all *Phalaenopsis* genes generated in this study red. Numbers above the nodes represent bootstrap values.

***DIV* clade 2**

***DIV* clade 1**

***DIV* clade 3**


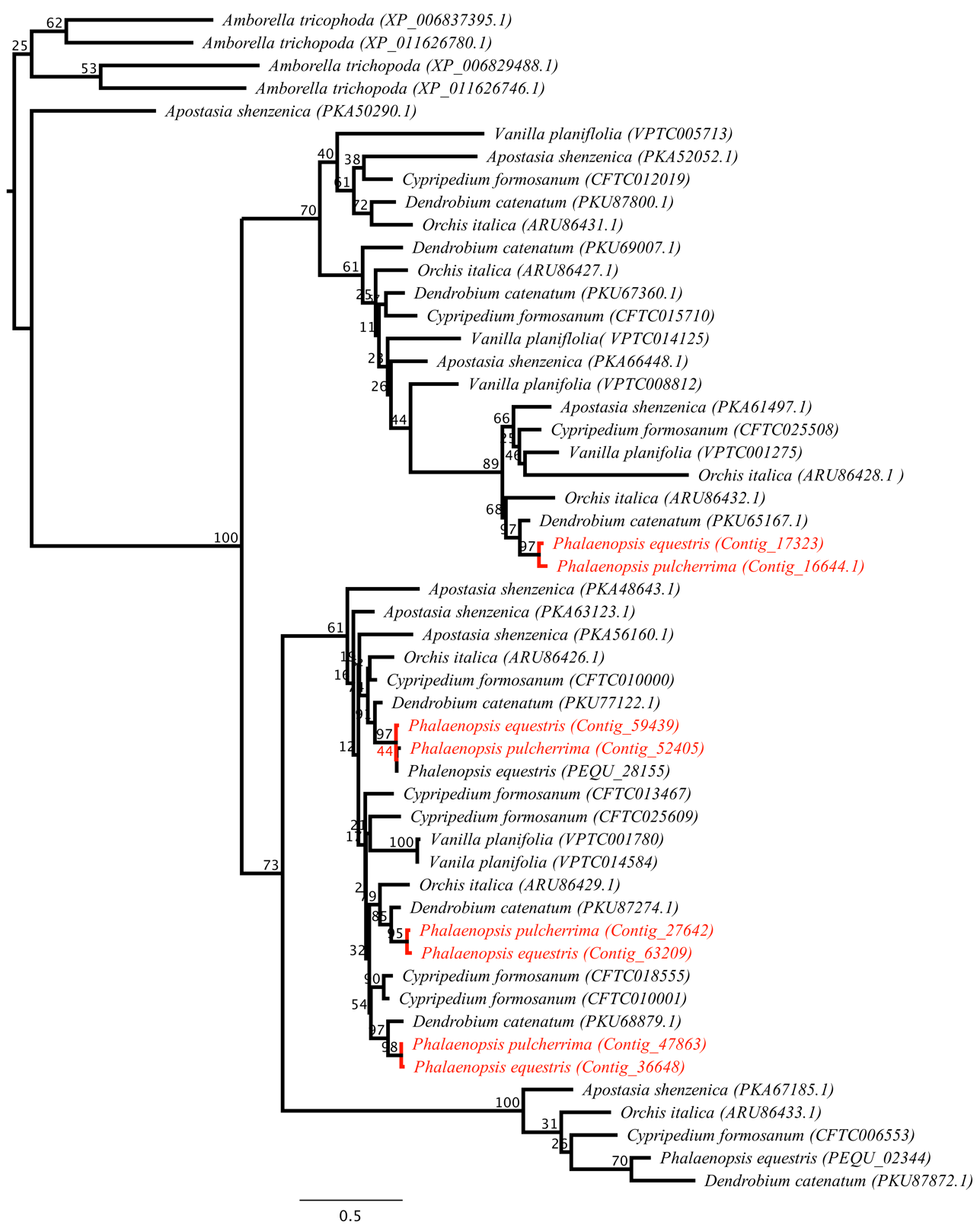


**Figure S8. Maximum likelihood tree of the DIV subfamily.** The phylogenetic tree was constructed with 100 bootstrap replicates based on the alignment of full-length amino acid sequences of the genes and edited by colouring the nodes belonging to all *Phalaenopsis* genes generated in this study red. Numbers above the nodes represent bootstrap values.


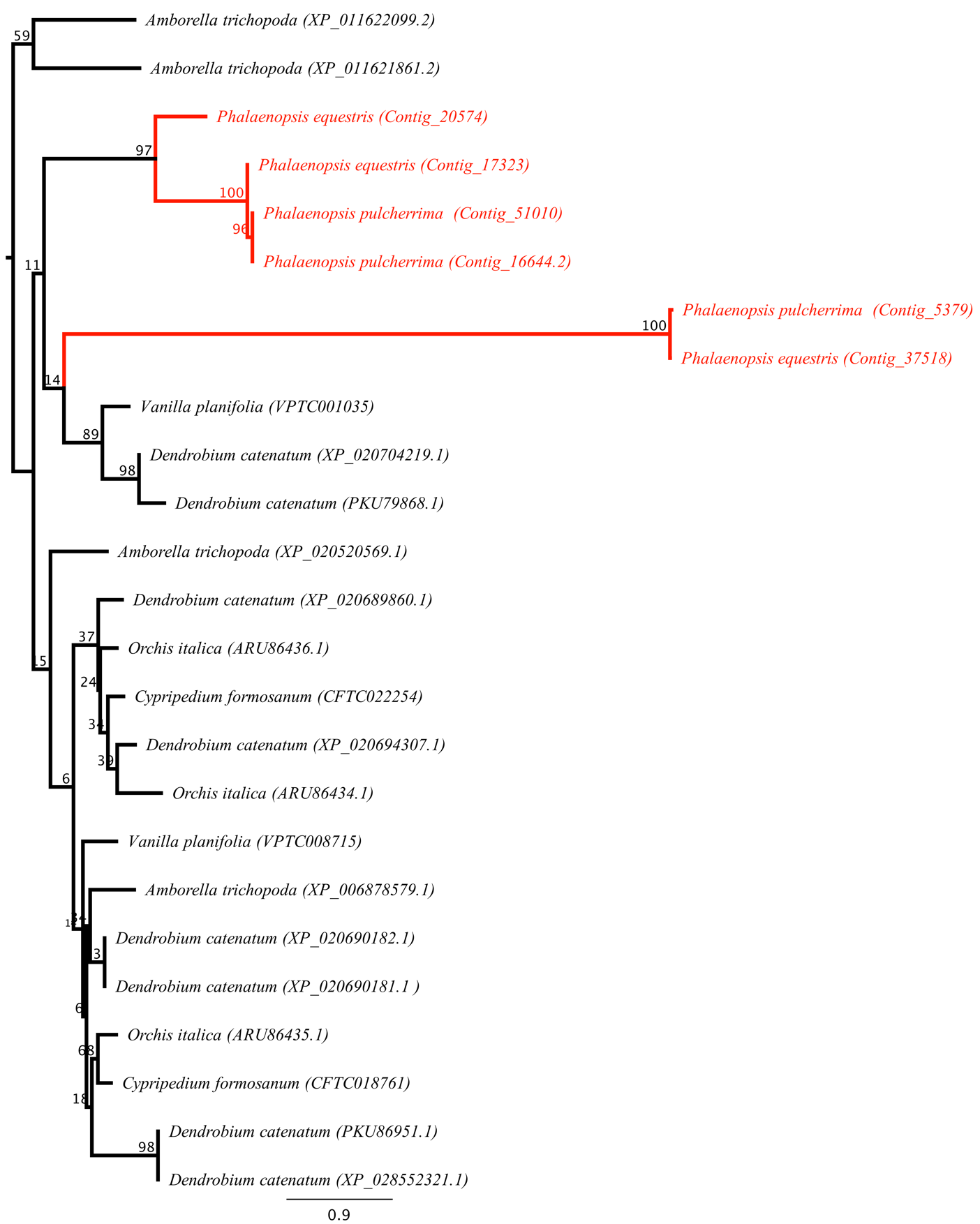


**Figure S9. Maximum likelihood tree of the RAD subfamily.** The phylogenetic tree was constructed with 100 bootstrap replicates based on the alignment of full-length amino acid sequences of the genes and edited by colouring the nodes belonging to all *Phalaenopsis* genes generated in this study red. Numbers above the nodes represent bootstrap values.

***DRIF* clade 1**

***DRIF* clade 3**

***DRIF* clade 2**


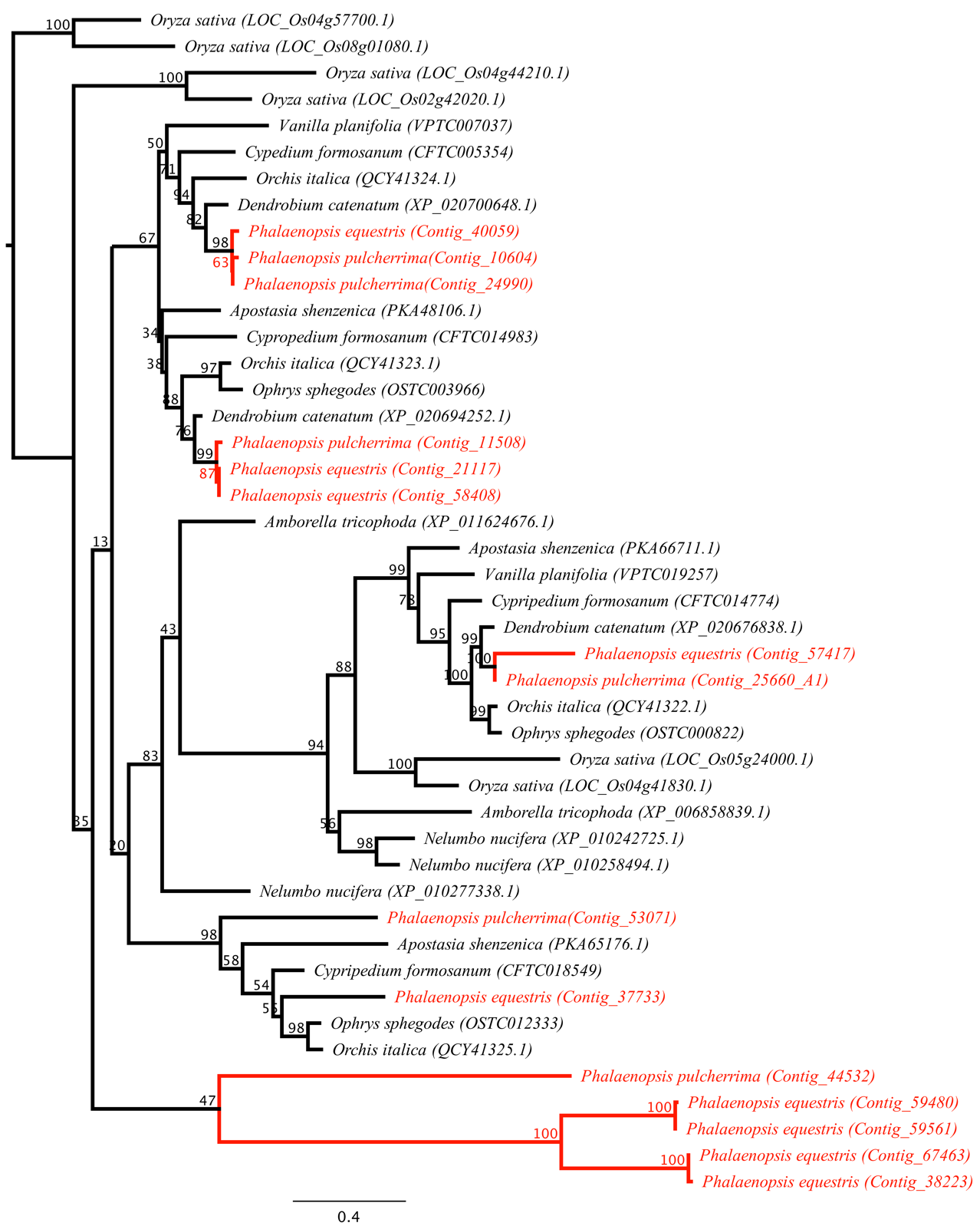


**Figure S10. Maximum likelihood tree of the DRIF subfamily.** The phylogenetic tree was constructed with 100 bootstrap replicates based on the alignment of full-length amino acid sequences of the genes and edited by colouring the nodes belonging to all *Phalaenopsis* genes generated in this study red. Numbers above the nodes represent bootstrap values.

***CIN* clade 1**

***PCF* clade 2**

***CYC/TB1* clade 3**

***CYC/TB1* clade 1**

***CIN* clade 2**

***PCF* clade 1**

***CYC/TB1* clade 2**


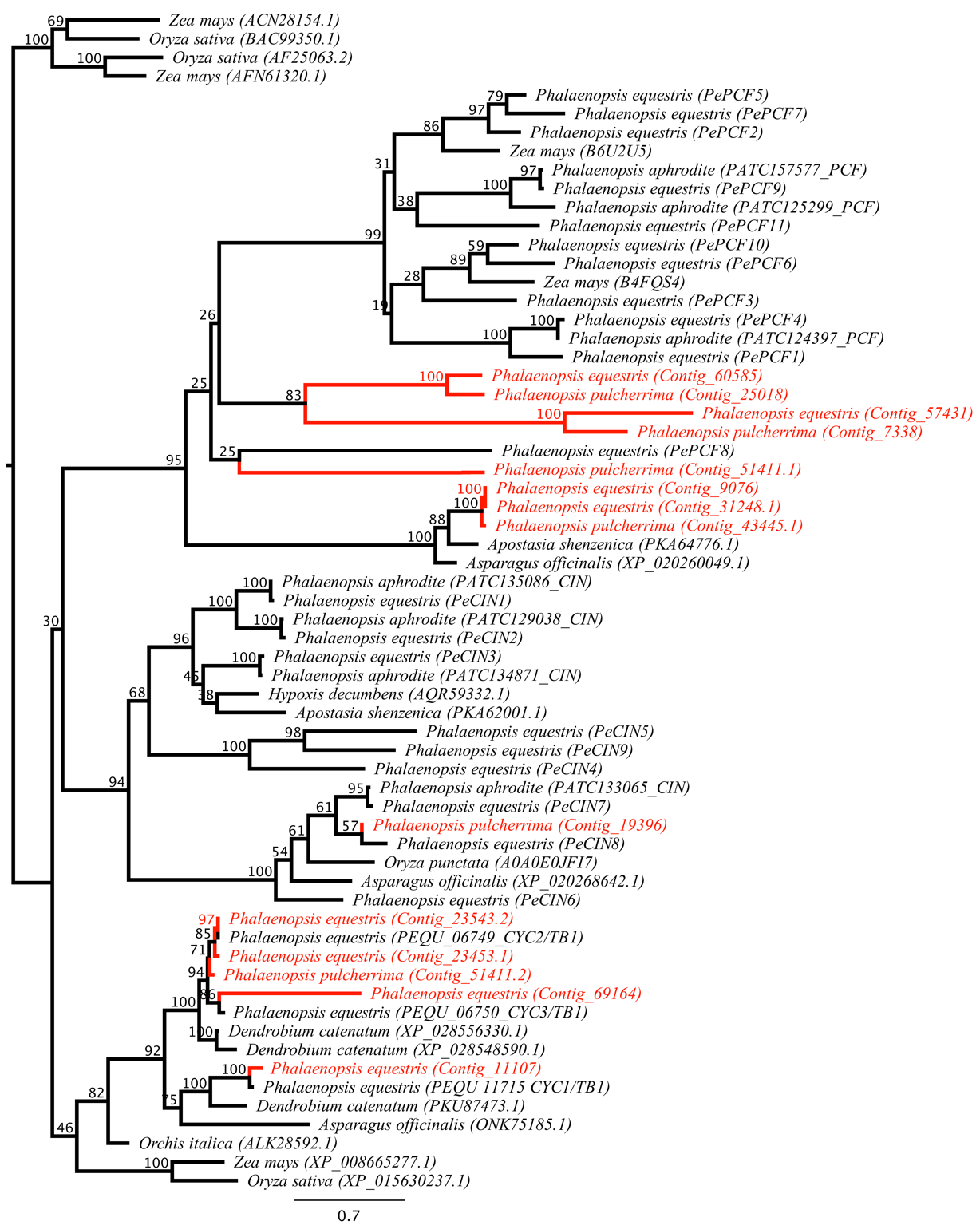


**Figure S11. Maximum likelihood tree of the TCP family.** The phylogenetic tree was constructed with 100 bootstrap replicates based on the alignment of full-length amino acid sequences of the genes and edited by colouring the nodes belonging to all *Phalaenopsis* genes generated in this study red. Numbers above the nodes represent bootstrap values.
